# American palm ethnomedicine: A meta-analysis

**DOI:** 10.1186/1746-4269-5-43

**Published:** 2009-12-24

**Authors:** Joanna Sosnowska, Henrik Balslev

**Affiliations:** 1 W. Szafer Institute of Botany, Polish Academy of Sciences, Lubicz 46, 31-512 Cracow, Poland; 2 Ecoinformatics and Biodiversity, Department of Biological Sciences, Aarhus University, Build. 1540, Ny Munkegade 114, DK-8000 Aarhus C., Denmark

## Abstract

**Background:**

Many recent papers have documented the phytochemical and pharmacological bases for the use of palms (*Arecaceae*) in ethnomedicine. Early publications were based almost entirely on interviews that solicited local knowledge. More recently, ethnobotanically guided searches for new medicinal plants have proven more successful than random sampling for identifying plants that contain biodynamic ingredients. However, limited laboratory time and the high cost of clinical trials make it difficult to test all potential medicinal plants in the search for new drug candidates. The purpose of this study was to summarize and analyze previous studies on the medicinal uses of American palms in order to narrow down the search for new palm-derived medicines.

**Methods:**

Relevant literature was surveyed and data was extracted and organized into medicinal use categories. We focused on more recent literature than that considered in a review published 25 years ago. We included phytochemical and pharmacological research that explored the importance of American palms in ethnomedicine.

**Results:**

Of 730 species of American palms, we found evidence that 106 species had known medicinal uses, ranging from treatments for diabetes and leishmaniasis to prostatic hyperplasia. Thus, the number of American palm species with known uses had increased from 48 to 106 over the last quarter of a century. Furthermore, the pharmacological bases for many of the effects are now understood.

**Conclusions:**

Palms are important in American ethnomedicine. Some, like *Serenoa repens *and *Roystonea regia*, are the sources of drugs that have been approved for medicinal uses. In contrast, recent ethnopharmacological studies suggested that many of the reported uses of several other palms do not appear to have a strong physiological basis. This study has provided a useful assessment of the ethnobotanical and pharmacological data available on palms.

## Background

Palms (*Arecaceae*) are common throughout the American tropics. They abound in hot, wet regions on the continents and associated archipelagos, particularly in areas covered by tropical rainforests [1,2]. Most of the 730 species of American palms [3] are used, particularly in rural areas, for food, shelter, fuel, medicine, and many other purposes [4]. For instance, in Ecuador, uses have been recorded for 111 of the 123 palm species present in that country [5]. This study extends from Plotkin and Balick's (1984) seminal paper that reviewed the medicinal uses of American palms. Plotkin and Balick deplored that the biodynamic and organic ingredients of palms were nearly unknown; but, during the quarter century since that publication, there has been increasing interest in palms as a source of active compounds. In recent years, pharmacological studies have become more numerous than ethnobotanical reports on medicinal palms. This may help bridge the gap between ethnobotanical data and clinical testing for the beneficial effects of palm products on human health.

The primary aim of this bibliographical survey was to compare existing ethnobotanical and pharmacological studies on the medicinal uses of palms. Gathering this large quantity of information may facilitate future search for new palm-derived medicines. A preliminary version of our database was presented at a palm symposium in Lima, Peru, in 2007 [6]. For this paper, we expanded the database with information from new and additional references and we present a full analysis of the data.

## Methods

We collected literature reports concerning the use of American palms in ethnomedicine and organized them according to palm species and use categories. We updated the nomenclature of palm names, including the authors' names, according to the World Checklist of Palms [7]. Thus, when the name of a palm in a publication was considered a synonym of an accepted name, we used the accepted name. Plotkin and Balick (1984) listed the medicinal uses of palms under each species name. Instead, we presented the information according to categories of health disorders, in accordance with the Economic Botany Data Collection Standard prepared for the International Working Group on Taxonomic Databases for Plant Sciences [8]. However, we departed from this standard by subdividing infections/infestations into the following categories: Fever, Bacterial, Fungal, Parasitic, and Viral infections. We added another category for Social Uses, including healing rituals, smoking materials, intoxicants, and religious uses that often influence human health (See additional file 1 for the categorized data derived from our literature searches). The latter categories are not included in the medicinal categories in Cook (1995).

Palms provide several nutritious food items. Therefore, they may be important in combating nutrient deficiency, a serious public health problem, particularly in developing countries. The nutrient compositions of palm fruits and palm-cabbage have been widely investigated and reported in the literature; therefore, these are not reviewed here.

**Figure 1:**
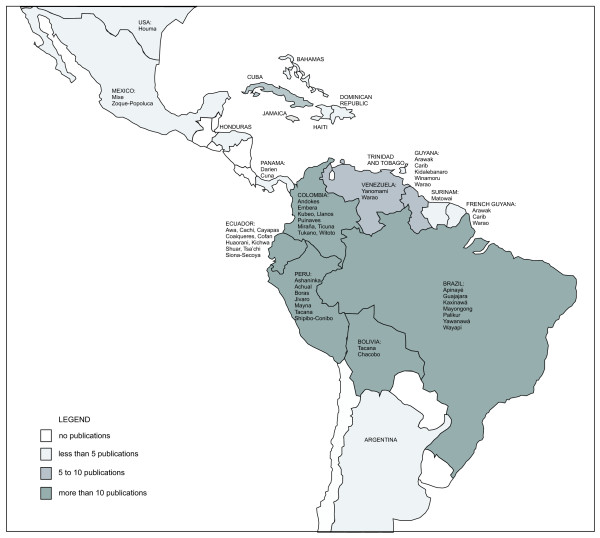
**Study area indicating the amounts of reviewed publications and ethnic groups per country**.

Publications that described the use of palms in ethnomedicine covered most American countries where palms are found (Fig. 1). In addition, they investigated many, but far from all, ethnic groups in tropical America. The papers reviewed here cover both indigenous and non-indigenous societies.

Our bibliographic searches employed several databases, including PubMed, Embase, and RedLightGreen. In addition, we conducted dedicated searches with the search engines of The State and University Libraries of Aarhus. We also accessed many publications in the reprint collections accumulated by one of the authors (HB) over many years of palm and ethnobotanical research. We performed special searches at the Amazonian Library and in the Instituto de Investigaciones en la Amazonía Peruana (IIAP) in Iquitos, Peru.

## Results and Discussion

### Medicinal palms

**Figure 2:**
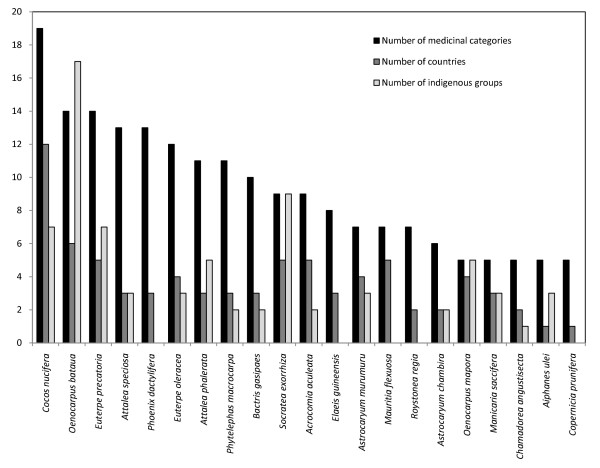
**Numbers of medicinal categories, indigenous groups, and countries involved in the use of individual American palm species**.

We found reports of medicinal uses for 106 American palm species (see Additional File 1). The most commonly used were: *Cocos nucifera*, for 19 different medicinal use categories, *Oenocarpus bataua*, for 14 categories; and *Euterpe precatoria*, for 14 categories (Fig. 2). *Cocos nucifera *is commonly cultivated throughout the Americas and was most likely introduced from other countries; in contrast, *O. bataua *and *E. precatoria *are native to the tropical rainforests of the Amazon basin and adjacent regions. The next most commonly used species was *Attalea speciosa *(13 categories). *Phoenix dactylifera, Euterpe oleracea, Attalea phalerata, Phytelephas macrocarpa, Bactris gasipaes, Acrocomia aculeata*, and *Socratea exorrhiza *were employed in 9-12 different medicinal use categories. Among American indigenous peoples, the best-known ethnomedicinal palms are the native palms, *O. bataua, S. exorrhiza, E. precatoria*, and a species with unknown origin, *C. nucifera *[1]. *C. nucifera *is widely cultivated throughout the tropical areas of the world. Thus, medicinal use of *C. nucifera *is the most widespread of the palms in the Americas (Fig. 2).

**Figure 3:**
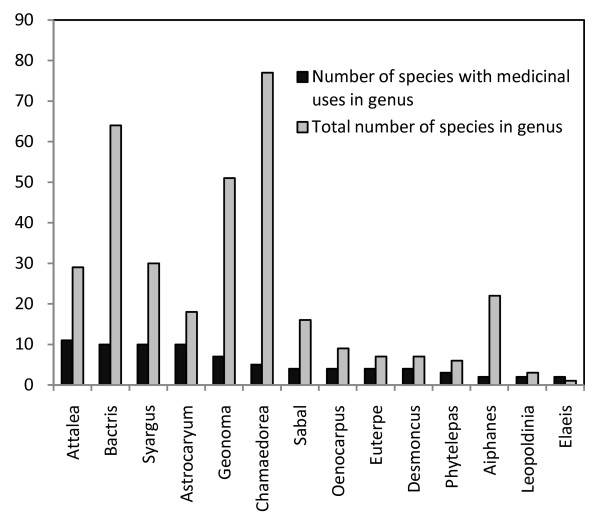
**American palm genera in which several species are used medicinally**.

The fruit of the palm is most commonly used for medicinal purposes; the fruit is used in 56 species, the oil is used in 19 species, the mesocarp in 16 species, and the endosperm in 11 species. Other parts of the palms that are used include roots (27 spp.), leaves (22 spp.), palm hearts (19 spp.), stems (17 spp.), and flowers (9 spp.). Often, several species in the same genus are used medicinally (Fig. 3). For example, the genus *Attalea *has 11 different species with medicinal uses; the genera *Astrocaryum*, *Bactris*, and *Syagrus *each have 10 medicinal species; and the genus *Geonoma *has seven medicinal species; all the other genera have 1-5 species that are used medicinally. As expected, species from the same genus often have similar uses [9] but there are exceptions to this rule; *O. bataua *prevents fever, but *Oenocarpus bacaba *can be harmful to a person recovering from intermittent fever [10,11].

Obviously, most medicinal uses of palms are beneficial; but, like many other drugs, inappropriate use may be harmful. For instance, a beverage made of *O. bataua *fruit is said to quickly cause a "horrible death" when mixed with liver of tapir [12]. The prevalence of medicinal palm use is notable; for example, the roots of *E. precatoria *are used medicinally almost everywhere it grows [10,13-19].

### Categories of health disorders treated with palms

**Figure 4:**
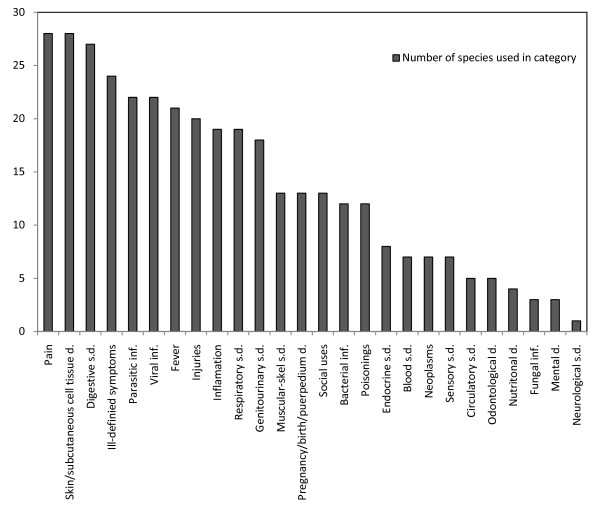
**Categories in which American palms are used to treat medicinal ailments**. s.d. = system disorders; inf. = infections.

In a general ethnomedicinal study of indigenous communities in Mexico, the most frequent ailments treated with medicinal plants were gastrointestinal disorders, dermatological diseases, and respiratory disorders [20]. These categories were also confirmed by other authors [21-23] that studied ethnomedicines in the indigenous societies of the Americas. In this paper, we used a different classification of diseases [8] (Fig. 4); our data related mainly to indigenous, but also to some non-indigenous peoples; and we looked only at health conditions treated with medicines from a single plant family. Among the diseases treated by palms, digestive system disorders were frequent, but pain ailments and skin tissue disorders were even more frequent. The preponderance of pain, injuries, and muscular-skeletal system disorders that could be treated with palm medicines may have reflected the epidemiological characteristics of the indigenous peoples in the region. Some indigenous peoples live in a traditional way, where hunting and gathering play a considerable role in subsistence. Hence, injuries and muscular-skeletal system disorders may be directly connected to everyday hunting activities. Palm-derived remedies may provide emergency treatments, as they commonly grow around villages and are easily accessible. Emergency medicines derived from palms, particularly those with styptic properties, like those derived from *A. speciosa*, are commonly used by the Apinayé and Guajajara Indians of northeastern Brazil [12]. Among the Warao and Arawak of Guyana, *E. oleracea *is considered a useful hemostatic medicine when a person is injured deep in the forest [24]; the sap of *Euterpe edulis *also possesses hemostatic properties [25].

Circulatory system disorders are among the major health problems in Western civilization; however, these seem not to be important among peoples that use American ethnomedicines derived from palms. Similarly, there are almost no reports of neurological system disorders that are common among ageing Western societies.

### Indirect impact of palm on human health

Palm medicines also have an indirect influence human health. Beetle larvae that live in the decaying stems of *Astrocaryum chambira, Wettinia maynensis *[26], *A. phalerata*, and *O. bataua *are collected and used in medicines to cure severe chest pains [27-29]. A fungus that causes chromoblastomycosis has been found in the shell of *A. speciosa*, which may explain the prevalence of this disease among farm workers in the Amazon region of Maranhao in Brazil [30]. Problems with *Triatominae *infested palms are common in many places [31-41]. Most *Rhodonius *sp. (*Hemiptera, Reduviidae, Triatominae*) are associated with palm trees; they transmit *Trypanosoma cruzi*, the organism responsible for Chagas disease throughout the American tropics [42].

Traditional medicines of American peoples are also used to treat some folk illnesses like *susto *- bad spirit. Folk illnesses form part of the broader indigenous world-view, and there are no direct equivalents in modern medicine nomenclature. From the medical point of view, folk illnesses are not diseases *sensu stricto*, but clusters of loosely bound syndromes. Consequently, in the category of Social Uses for palm medicines, we included uses connected to healing rituals and folk illnesses.

### Local *versus *pharmacological knowledge

As mentioned above, previous knowledge about the ethnomedicinal uses of American palms was based primarily on local knowledge and ethnobotanical studies [25]; in contrast, many recent papers have investigated the phytochemical and pharmacological bases for ethnomedicinal treatments. We compared local rationale and pharmacological evidence in a number of cases where both kinds of evidence existed.

There are many, often conflicting, reports about the medicinal properties of *A. speciosa *(babassu). Mesocarp flour from its fruit is used in Brazil to treat pain, constipation, obesity, leukemia, rheumatism, tumors, and ulcerations (references in Additional File 1). It has also been shown to have anti-inflammatory [43] or pro-inflammatory [44] properties, depending on the inflammation model and the time or route of treatment. These reports, together with the fact that it is used to treat venous disease, suggest that it could be a good candidate for the development of new medicines [45]. However, when tested, extracts of its leaves, flowers, and endocarp did not show pharmacological activity [46]. This may suggest that the uses are based on physiological effects of the mesocarp, which may have properties not found in other parts of babassu palm.

Interestingly, sap from the stem of *E. oleracea *(açai) is used as a hemostatic agent among Matowai, Arawak, and Warao peoples; however, pharmacological studies indicated that the fruit stone extract of *E. oleracea *had a vasodilator effect [47]. The fruit of *E. oleracea *also holds promise for use in modern medicine as an alternative oral contrasting agent for MRI (Magnetic Resonance Imaging) studies of the gastrointestinal system [48].

The traditional claim that roots of *E. precatoria *have anti-inflammatory activity appears to have been verified, although the mechanism that underlies the effect remains unknown [19].

The roots of *E. precatoria *and the fruits of *O. bataua *were traditionally and are presently used to treat malaria in Peru [[15], Rengifo 2007, and Agencia Española de Cooperación Internacional para el Desarollo 2007 personal communication]. Although Deharo et al. (2001) [49] did not include these species in their pharmacological tests of anti-malarial activity, other studies showed a moderate antiplasmodial activity in extracts of *E. precatoria *roots [50].

In ethnobotanical studies, the fruit extracts of *Astrocaryum vulgare *and *A. speciosa *have been reported to be useful for the treatment of skin diseases. However, when they were screened for anti-tyrosinase activity, the effect was poor [51].

Hypoglycemic compounds have been found in the roots of *A. aculeata *in many studies; this coincides well with its traditional use as a treatment for diabetes among indigenous peoples in Mexico [52-54].

Coconut (*C. nucifera*) oil was shown to have antiseptic effects and is used as an efficient, safe skin moisturizer [55]. This suggested that its traditional use as a lotion in many parts of America is well founded. Its selective antibacterial effects [56] also make it useful for topical applications in wound healing. Other studies have suggested that extracts from *C. nucifera *husk fibers could be useful in the treatment of leishmaniasis [57,58]. In traditional Mexican medicine, *C. nucifera *has been used to treat trichomoniasis, dysentery, and enteropathogens. These uses are consistent with its antimicrobial effects. Moreover, recent pharmacological studies have demonstrated that extracts of *C. nucifera *could be used as an alternative method to treat drug resistant enteric infections [59,60]. In vivo assays demonstrated that *C. nucifera *extract had low toxicity, and that it did not induce dermic or ocular reactions [57]. Thus, considering its potent antioxidant activity and low toxicity, husk fiber extracts of *C. nucifera *have potential in the treatment of oral diseases [61]. Furthermore, its aqueous extract may be a source of new drugs with anti-neoplastic and anti-multidrug resistance activities [62]. It is of great interest for cancer therapy to identify new compounds that are able to overcome resistance mechanisms and lead to tumor cell death.

Seeds of *Aiphanes aculeata *were traditionally used for treatment of cancer by people from the Peruvian Amazon region (Rengifo, personal communication, 2007). Lee et al. (2001) [63] isolated Aiphanol and some other compounds that may have chemopreventive properties, but they have not been pharmacologically tested for their effects on cancer.

Increasing attention has focused on the use of phytotherapeutic agents to treat prostate disorders, ranging from benign prostatic hyperplasia to prostate cancer. The best described phytotherapeutic agent for prostate disorders was from *Serenoa repens *(saw palmetto) [[64-68], among others]. Several studies have reported no side effects of saw palmetto treatments [69], and a dietary supplement with *S. repens *may be effective in controlling prostate cancer tumorigenesis [70]. The extract of *S. repens *was as effective as the tested pharmaceuticals in the relief of urinary symptoms [71,72]. The roots of *Roystonea regia *are used as a traditional medicine in Eastern Cuba to cure impotence [73]. Currently, *R. regia *fruit lipid extracts are being tested in the Cuban National Center for Scientific Research, and preliminary results suggest that they appear to provide a promising treatment for prostatic hyperplasia [74-82].

## Conclusions

Palms have been used for the treatment of various human ailments throughout the Americas by many societies and in many regions. Local knowledge about the medicinal uses of palms is extensive, and recent ethnopharmacological studies have confirmed the effectiveness of many of the treatments; however, some uses appear not to have a physiological basis and others have not been investigated.

Ethnopharmacological research may improve the therapeutic use of traditional medicine in regions where these palms were originally used, and this information may also be important for the design of new drugs. Further analysis of the pharmacological activities of palm extracts may enable the design of less expensive therapies. Natural products are currently the leading source of new biologically active compounds. The ingestion of palm products with medicinal properties represents a concrete alternative treatment in many areas with limited access to modern medicine. Pharmacological analyses have previously focused on the antibiotic effects of palm medicines, but these medicines have also shown potential in the fight against prostatic hyperplasia, diabetes, leishmaniasis, and other diseases.

## List of abbreviations

own obs: own observation; BACTER: bacterial infections; BLOOD: blood system disorders; CIRCUL: circulatory system disorders; DIGEST: digestive system disorders; ENDOCR: endocrine system disorders; FUNGAL: fungal infections; GENITO: genitourinary system disorders; ILLDEF: ill-defined symptoms; INFLAM: inflammations; INJUR: injuries; MENTAL: mental disorders; MUSCUL: muscular-skeletal system disorders; NEUROL: neurological system disorders; NEOPL: neoplasms; NUTRIT: nutritional disorders; ODONT: odontological disorders; PARASI: parasitic infections; POISON: poisonings; PREGN: pregnancy/birth/puerperium disorders; RESPIR: respiratory system disorders; SENSOR: sensory system disorders; SKIN: skin/subcutaneous cell tissue disorders; SOCIAL: social uses; VIRAL: viral infections.

## Competing interests

The authors declare that they have no competing interests.

## Authors' contributions

The article was initiated by JS, who searched the literature and made the preliminary report on medicinal palms. The manuscript was prepared by JS and HB. Both authors have read and approved the final version of the manuscript.

## Supplementary Material

Additional file 1 **Clinical categories for disorders that are treated with use of American palms**. This table provides a comprehensive list of the data collected on ethnomedicines derived from American palms. The data are organized by ailment, and includes: the associated disease, the palm species, the way the medicine is prepared, the part of the palm that is used, the country that uses the ethnomedicine, the region of that country, the indigenous group of people that use the medicine, and the reference for the finding [83-151]. Click here for file
