# Supplementary material for: American palm ethnomedicine: A meta-analysis
Source: J Ethnobiol Ethnomed. 2009 Dec 24;5:43. doi: 10.1186/1746-4269-5-43 (PMC2804589; doi:10.1186/1746-4269-5-43)
Supplement: Additional file 1 — Clinical categories for disorders that are treated with use of American palms. This table provides a comprehensive list of the data collected on ethnomedicines derived from American palms. The data are organized by ailment, and includes: the associated disease, the palm species, the way the medicine is prepared, the part of the palm that is used, the country that uses the ethnomedicine, the region of that country, the indigenous group of people that use the medicine, and the reference for the finding [83-151]. [file 1746-4269-5-43-S1.PDF]

Table 1. Clinical categories for disorders that are treated with use of American palms

This table provides a comprehensive list of the data collected on ethnomedicines derived from American palms. The data are organized by ailment, and includes: the associated disease, the palm species, the way the medicine is prepared, the part of the palm that is used, the country that uses the ethnomedicine, the region of that country, the indigenous group of people that use the medicine, and the reference for the finding.

**Medicinal category**	**Disease**	Species	**Way of preparation**	**Part used**	**Country, region**	**Indigenous group**	**Reference**
BACTER.	dysentery	*Attalea phalerata*	decoction made to syrup together with *O. bataua*	root, young and red	Bolivia	Tacana	DeWalt et al. 1999
BACTER.	tuberculosis	*Attalea phalerata*	internally	fruit, oil	Bolivia	Tacana	DeWalt et al. 1999
BACTER.	dysentery	*Attalea speciosa*	internally	root	Bolivia	Tacana	UMSA et al. 1999
BACTER.	dysentery	*Euterpe precatoria*	decoction made to syrup	root, young and red	Bolivia	Tacana	DeWalt et al. 1999
BACTER.	dysentery	*Oenocarpus bataua*	together with *A. phalerata* into syrup	root	Bolivia	Tacana	DeWalt et al. 1999
BACTER.	erysipelas	*Astrocaryum aculeatissimum*		fruit	Brazil		[Usher 1974, nv], Plotkin and Balick 1984
BACTER.	erysipelas	*Attalea oleifera*		fruit, seed oil	Brazil		[Pereira 1929 nv], Plotkin and Balick 1984
BACTER.	dysentery	*Cocos nucifera*		root	Colombia		Garcia Barriga 1974
BACTER.	veneral diseases	*Cocos nucifera*		root, young	Colombia		Garcia Barriga 1974
BACTER.	tuberculosis	*Oenocarpus bataua*	internally	fruit, mesocarp oil	Colombia, Bolivia, Peru, Ecuador	Witoto, Llanos, Tacana/ Kichwa, all Amazonian natives	[Schultes 1951 nv], Garcia Barriga 1974, Perez-Arbelaez 1978, Plotkin and Balick 1984, Balick 1986, Balick 1989, Schultes, Raffauf 1990, Kahn and Granville 1992, DeWalt et al. 1999, Bennett et al. 2002, Diaz 2003, Rengifo

							2007, Torre et al. 2007
BACTER.	antibacterial	*Cocos nucifera*	herbal mixture with *Lepidium virginicum* decoction, internally	fruit, root	Cuba		Cano and Volpato 2004
BACTER.	veneral diseases	*Cocos nucifera*	decoction in herbal mixtures, orally	root, fruits young	Cuba		Cano and Volpato 2004
BACTER.	veneral diseases	*Roystonea regia*	decoction in herbal mixtures, orally	root	Cuba		Cano and Volpato 2004
BACTER.	gonorrhea	*Cocos nucifera*		fruit, endosperm	Ecuador	Afroecuatorian	Torre et al. 2007
BACTER.	dysentery	*Mauritia flexuosa*	sago-like pap prepared from	stem, pith	Guyana		[Fanshawe 1950 nv], Plotkin and Balick 1984
BACTER.	dysentery	*Cocos nucifera*		fruit, exocarp	Mexico		Alanís et al. 2005
BACTER.	veneral diseases	*Euterpe precatoria*			Peru, Lower Amazon		Rengifo 2007
BACTER.	dysentery	*Dypsis lutescens*			Trinidad and Tobago		Ayensu, 1981
BACTER.	gonorrhea	*Cocos nucifera*		coconut water	West Indies		Ayensu, 1981
BACTER.	veneral diseases	*Astrocaryum campestre*	macerated in water, the concoction is left outside the house at night, and consumed early the next morning	leaf			Balick 1986
BACTER.	antibacterial	*Cocos nucifera*	aqueous extract	fruit, husk fiber			Esquenazi et al. 2002
BACTER.	antibacterial	*Cocos nucifera*	extract	fruit, husk fiber			Esquenazi et al. 2002, Alanis et al. 2005

BACTER.	cholera	*Cocos nucifera*		fruit, endosperm, liquid			Grimwood, 1975
BLOOD	anemia	*Attalea phalerata*	decoction-syrup, internally	root, young and red	Bolivia	Tacana	DeWalt et al. 1999, UMSA *et al.* 1999, Bourdy et al. 2000
BLOOD	anemia	*Euterpe precatoria*	decoction-syrup, internally	root, oil	Bolivia, Brazil, Peru	Tacana, Yawanawa, Kaxinawa	DeWalt et al. 1999, Bourdy et al. 2000, Campos and Ehringhaus 2003,Rengifo 2007
BLOOD	depurative	*Copernica prunifera*		root	Brazil		[Braga 1960 nv], Plotkin and Balik 1984, Balick 1989
BLOOD	depurative	*Desmoncus polyacanthos*		root	Brazil		[Pio Correa nv, LeCointe 1934 nv], Plotkin and Balick 1984, Balick 1989
BLOOD	anemia	*Cocos nucifera*	internally	fruit, endosperm, liquid	Colombia, Brazil		Perez-Arbelaes 1978, Campos and Ehringhaus 2003
BLOOD	depurative	*Roystonea regia*	decoction in herbal mixtures, internally	root	Cuba		Cano and Volpato 2004
BLOOD	anemia	*Acrocomia aculeata*			Mexico	Zoque-Popoluca	Leonti at al. 2003
CIRCUL.	venous diseases	*Attalea speciosa*	extract	fruit, mesocarp	Brazil	Apinaye, Guajajara	Azevedo et al. 2007
CIRCUL.	cardiovascular diseases	*Euterpe oleracea*	extract	fruit	Brazil		Matheus et al. 2006, Rocha et al. 2007
CIRCUL.	hemorroids	*Cocos nucifera*	decoction, herbal mixtures, orally	fruit	Cuba		Cano and Volpato 2004
CIRCUL.	hypertension	*Cocos nucifera*		fruit	French Guyana	Carib	Andel 2000a
CIRCUL.	cerebral stroke	*Elaeis guineensis*			Peru, Amazon		Rengifo 2007
CIRCUL.	hypertension	*Sabal sp.*			USA, Louisianna	Houma	Johnson 1999

DIGEST.	liver ailments	*Astrocaryum murumuru*	decoction, internally	root	Bolivia	Tacana	DeWalt et al. 1999
DIGEST.	stomachic	*Attalea phalerata*	decoction	root	Bolivia	Tacana	DeWalt et al. 1999
DIGEST.	diarrhea	*Attalea phalerata*	decoction, internally	root	Bolivia	Tacana	UMSA et al. 1999, Bourdy et al. 2000
DIGEST.	liver ailments	*Attalea speciosa*	eaten daily	fruit, oil	Bolivia		Balick 1989
DIGEST.	vomits	*Chamaedorea angustisecta*	infusion, internally	flower	Bolivia	Tacana	UMSA et al. 1999, Bourdy et al. 2000
DIGEST.	diarrhea	*Attalea princeps*	shreded and boiled pinnae, cooled decoction is drunk	leaf	Bolivia, Beni	Chacobo	Boom 1987
DIGEST.	diarrhea	*Chamaedorea angustisecta*	infusion, internally	flower	Bolivia, Peru	Tacana	UMSA et al. 1999, Bourdy et al. 2000, Rengifo 2007
DIGEST.	liver ailments	*Euterpe precatoria*	decoction, internally	root, fruit oil	Bolivia, Peru	Tacana	DeWalt et al. 1999, Deharo et al. 2004
DIGEST.	laxative	*Astrocaryum aculeatissimum*		fruit, water from unripe	Brazil		[Pio Correa 1926 nv], Plotkin and Balick 1984
DIGEST.	constipation	*Attalea speciosa*		fruit, mesocarp	Brazil		Nascimento et al. 2005
DIGEST.	oral diseases	*Cocos nucifera*		fruit, husk fiber	Brazil		Alviano et al. 2008
DIGEST.	diarrhea	*Cocos nucifera*	internally	coconut water	Brazil		Campos and Ehringhaus 2003
DIGEST.	diarrhea	*Cocos nucifera*	decoction	fruit, husk fiber	Brazil		Esquenazi et al. 2002
DIGEST.	purgative	*Desmoncus orthacanthos*	decoction	root	Brazil		[Pio Correa 1926 nv.], Plotkin and Balick 1984
DIGEST.	diarrhea	*Syagrus comosa*	roasted	fruit, endosperm	Brazil		[Pio Correa 1926 nv], Plotkin and Balick 1984
DIGEST.	stomachic	*Syagrus comosa*	fresh	palm heart	Brazil		[Pio Correa 1926 nv], Plotkin and Balick 1984

DIGEST.	carminative	*Syagrus oleracea*	eaten fresh	palm heart	Brazil		[Pio Correa 1926 nv.], Plotkin and Balick 1984
DIGEST.	stomachic	*Syagrus oleracea*	eaten fresh	palm heart	Brazil		[Pio Correa 1926 nv.], Plotkin and Balick 1984
DIGEST.	stomachic	*Syagrus pseudococos*	macerated in cold water	palm heart	Brazil		[Pio Correa 1926 nv], Plotkin and Balick 1984
DIGEST.	diarrhea	*Euterpe oleracea*		fruit, oil (dark green)	Brazil, Manaus		[Prance and da Silva 1975 nv], Plotkin and Balick 1984
DIGEST.	laxative	*Bactris guineensis*	decoction	fruit	Colombia		[Garcia Barrige 1974 nv], Plotkin and Balick 1984
DIGEST.	laxative	*Cocos nucifera*	raw	fruit, mesocarp, oil	Colombia		Ayensu 1981, García Barriga 1974
DIGEST.	gastrointestinal irritation	*Cocos nucifera*	raw	fruit, endosperm	Colombia		Garcia Barriga 1974
DIGEST.	purgative	*Cocos nucifera*	decoction	fruit, mesocarp	Colombia		Garcia Barriga 1974
DIGEST.	digestive	*Mauritia flexuosa*	a refreshing drink	fruit	Colombia		Perez Arbelaz 1978, Plotkin and Baick 1984
DIGEST.	laxative	*Mauritia flexuosa*	infusion	fruit, mesocarp	Colombia		Perez-Arbelaez 1978
DIGEST.	laxative	*Phoenix dactylifera*	dried or dissolved	fruit	Colombia		Garcia Barriga 1974
DIGEST.	diarrhea	*Ammandra decasperma*	internally	fruit, endosperm	Ecuador	Huaorani	Macia 2004, Torre et al. 2007
DIGEST.	purgative	*Attalea colenda*		fruit	Ecuador	Tsa'chi	Torre et al. 2007
DIGEST.	diarrhea	*Bactris corossilla*	mixed with chili pepper	palm heart	Ecuador	Huaorani	Ceron and Ayala 1998
DIGEST.	diarrhea	*Bactris gasipaes*	decoction	root	Ecuador	Kichwa	Balslev et al. 1997, Torre et al. 2007
DIGEST.	liver ailments	*Cocos nucifera*		fruit	Ecuador	Kichwa	Torre et al. 2007

DIGEST.	diarrhea	*Oenocarpus bataua*		root adventitious	Ecuador	Huaorani	Plotkin and Balick 1984, Lescure et al. 1987, Schultes and Raffauf 1990, Kahn and Granville 1992
DIGEST.	stomach ailments	*Oenocarpus bataua*		root adventitious	Ecuador	Huaorani	Plotkin and Balick 1984, Lescure et al. 1987, Schultes and Raffauf 1990, Kahn and Granville 1992
DIGEST.	diarrhea	*Oenocarpus bataua*	liquid mixed with yuca	root	Ecuador	Kichwa	Torre et al. 2007
DIGEST.	gastritis	*Phytelephas aequatorialis*		fruit	Ecuador	Tsa'chi	Torre et al. 2007
DIGEST.	gall bladder ailments	*Euterpe precatoria*	infusion taken internally	root	Ecuador, Peru	Kichwa	AECID 2007, Torre et al. 2007
DIGEST.	diarrhea	*Oenocarpus bataua*	internally	fruit	Ecuador, Peru	Huaorani	Ceron and Ayala 1998, Rengifo 2007
DIGEST.	diarrhea	*Manicaria saccifera*	liquid jelly from young	fruit	French Guyana	Arawak	Andel 2000a,b
DIGEST.	diarrhea	*Mauritia flexuosa*	sago-like pap prepared from	stem, pith	Guyana		[Fanshawe 1950 nv], Plotkin and Balick 1984
DIGEST.	diarrhea	*Acrocomia aculeata*		fruit	Mexico	Zoque-Popoluca	Leonti et al. 2003
DIGEST.	dispepsia	*Thrinax radiata*			Mexico		Johnson 1999
DIGEST.	diarrhea	*Euterpe precatoria*	boiled and taken internally	root	Peru		AECID 2007
DIGEST.	laxative	*Oenocarpus mapora*	mixed with hot water	fruit, green	Peru	Bora	[Balick 1980 nv], Plotkin and Balick 1984
DIGEST.	nausea	*Oenocarpus mapora*	mashed taken internally	fruit green	Peru		Duke and Vasquez 1994
DIGEST.	nausea	*Oenocarpus minor*	mashed taken internally	fruit green	Peru		Duke and Vasquez 1994
DIGEST.	liver ailments	*Astrocaryum chambira*			Peru, Amazon		Rengifo 2007

DIGEST.	antiemetic	*Chamaedorea angustisecta*			Peru, Amazon		Rengifo 2007
DIGEST.	antiemetic	*Oenocarpus bataua*			Peru, Amazon		Rengifo 2007
DIGEST.	laxative	*Oenocarpus bataua*			Peru, Amazon		Rengifo 2007
DIGEST.	liver ailments	*Oenocarpus bataua*			Peru, Amazon		Rengifo 2007
DIGEST.	diarrhea	*Oenocarpus mapora*	mashed taken internally	fruit green	Peru, Amazon		Duke and Vasquez 1994, Rengifo 2007
DIGEST.	diarrhea	*Oenocarpus minor*	mashed taken internally	fruit green	Peru, Amazon		Duke and Vasquez 1994
DIGEST.	purgative	*Oenocarpus bataua*	mixed with sweet almond oil	fruit, oil	Peru, Ecuador	Kichwa	Plotkin and Balick 1984, Torre et al. 2007
DIGEST.	digestive	*Mauritia flexuosa*	preserved in leaves of *Asclepias curasavica,* internally	fruit, mesocarp	Venezuela		[Pittier 1926 nv], [Perez Arberalaez 1956], Plotkin and Balick 1984
DIGEST.	laxative	*Mauritia flexuosa*	preserved in leaves of *Asclepias curasavica,* internally	fruit, mesocarp	Venezuela		[Pittier 1926 nv], [Perez Arberalaez 1956], Plotkin and Balick 1984
DIGEST.	diarrhea	*Acrocomia aculeata*		fruit			Balick 1989
DIGEST.	laxative	*Attalea speciosa*					Balick 1988, García Barriga, 1992,
DIGEST.	stomachic	*Cocos nucifera*	coconut water itself or mixed with coconut milk	fruit			Grimwood 1975, García Barriga 1992, Heinrich 2000
DIGEST.	oral contrast agent for MRI of the gastrointestinal tract	*Euterpe oleracea*	orally or injected	fruit, mesocarp			Cordava-Fraga et al.. 2004

DIGEST.	stomachic	*Phoenix dactylifera*		palm heart			UMSA et al. 1999
ENDOCR.	diabetes	*Euterpe precatoria*	decoction, internally	root	Bolivia	Tacana	UMSA et al. 1999, Bourdy et al. 2000
ENDOCR.	diabetes	*Acrocomia aculeata*	charring	root	Mexico		Quero 1992, Perez et al. 1997, Haines 2004, Andrade-Cetto and Heinrich 2005
ENDOCR.	diabetes	*Phytelephas macrocarpa*		fruit	Peru		Mejia 1985
ENDOCR.	diabetes	*Euterpe oleracea*			Peru, Lower Amazon		Rengifo 2007
ENDOCR.	hypoglycemic effect	*Cocos nucifera*	dried	fruit, shells			Balick 1989
ENDOCR.	oestrogenic effects	*Cocos nucifera*		fruit, oil			Balick 1989
ENDOCR.	oestrogenic effects	*Elaeis guineensis*		seedlings			Balick 1989
ENDOCR.	oestrogenic effects	*Phoenix dactylifera*	dried	pollen and kernels			Balick 1989
ENDOCR.	antiestrogenic	*Serenoa repens*		fruit, seed			Di Silverio et al. 1992,
FEVER	fever	*Oenocarpus mapora*	mixed with water and drunk	stem sap	Bolivia, Beni	Chacobo	[Boom 1987] Milliken 1997
FEVER	fever	*Socratea exorrhiza*	boiled, cooled decoction is drunk	fruit, bark scrapings	Bolivia, Beni	Chacobo	[Boom 1987] Milliken 1997
FEVER	fever	*Attalea phalerata*	internally and externally	fruit, seed, oil	Bolivia, Madidi	Tacana	DeWalt et al. 1999, UMSA et al. 1999, Bourdy et al. 2000, Paniagua-Zambrana 2005
FEVER	fever	*Attalea phalerata*	mixed with root of *E. precatoria*	root	Bolivia, Madidi	Tacana	Paniagua-Zambrana 2005
FEVER	fever	*Attalea speciosa*		fruit, seed, oil	Bolivia, Madidi		Paniagua-Zambrana 2005
FEVER	fever	*Chamaedorea angustisecta*		flower	Bolivia, Madidi	Tacana	Paniagua-Zambrana 2005

FEVER	fever	*Oenocarpus bataua*	internally, externally	fruit, seed, oil	Bolivia, Madidi	Tacana	DeWalt et al. 1999, UMSA et al. 1999, Bourdy et al. 2000, Paniagua-Zambrana 2005
FEVER	fever	*Euterpe precatoria*	mixed with root of *A. phalerata,*infusion, internally	root	Bolivia, Peru		Paniagua-Zambrana 2005, AECID 2007
FEVER	cause fever	*Acrocomia aculeata*		fruit	Brazil		[Hoehne 1939] Plotkin and Balick 1984
FEVER	fever	*Allagoptera campestris*		fruit, bitter mesocarp	Brazil		[Pio Correa 1926 nv], Plotkin and Balick 1984
FEVER	fever, quench thirst	*Attalea spectabilis*	dissolved in water	fruit, endocarp	Brazil		[Pio Correa 1926, nv], Plotkin and Balick 1984
FEVER	fever	*Desmoncus mitis*		root	Brazil	Kaxinawa	Campos and Ehringhaus 2003
FEVER	fever	*Phytelephas macrocarpa*		leaf, young	Brazil	Kaxinawa	Campos and Ehringhaus 2003
FEVER	fever	*Euterpe oleracea*	roasted and made in coffe-like beverage	fruit, seed	Brazil, Peru		Duke and Vasquez 1994, Milliken 1997, Matheus et al. 2006, Rengifo 2007
FEVER	typhoid fever	*Cocos nucifera*			Cuba		Cano and Volpato, 2004
FEVER	fever	*Chamaedorea pinnatifrons*	alcohol extract mixed with other herbs	fruit	Ecuador	Shuar	Torre et al. 2007
FEVER	fever	*Cocos nucifera*		flower, root	Ecuador	Afroecuatorian	Torre et al. 2007
FEVER	fever	*Astrocaryum chambira*			Peru, Amazon		Rengifo 2007
FEVER	fever	*Cocos nucifera*	decoction drunk	root	Venezuela		Milliken 1997
FEVER	fever	*Manicaria saccifera*	juice of young strained and drunk 3 per day	fruit	Venezuela	Warao	Milliken 1997, Plotkin and Balick 1984
FEVER	malarial fever	*Euterpe precatoria*	decoction from crushed and boiled	root		Peru, Lower Amazon	Kahn and Granville 1992, Rengifo 2007

FEVER	fever	*Mauritia sp.*				Warao	Plotkin and Balick 1984
FEVER	fever	*Phoenix dactylifera*					Johnson, 1999
FUNGAL	athlete foot	*Oenocarpus bataua*		fruit, oil	Ecuador	Kichwa	Torre et al. 2007
FUNGAL	thrush	*Astrocaryum gynacanthum*	ash rubbed on mouth sores	leaf	Guyana	Arawak, Carib	Andel 2000a,b
FUNGAL	thrush	*Manicaria saccifera*	milk of young	fruit	Guyana		Plotkin and Balick 1984
GENITO	amenorrhea	*Cocos nucifera*	infusion, internally	root	Jamaica, Bahamas, Exuma and Long Islands		Ayensu, 1981
GENITO	menorrhagia	*Cocos nucifera*		fruit, endosperm	Jamaica, Bahamas, Exuma and Long Islands		Ayensu, 1981
GENITO.	kidney ailments	*Astrocaryum murumuru*	decoction, taken internally	root	Bolivia	Tacana	DeWalt et al. 1999, Bourdy et al. 2000
GENITO.	irregular menses	*Chamaedorea angustisecta*	toasted, infusion	flower	Bolivia	Tacana	UMSA et al. 1999, Bourdy et al. 2000
GENITO.	haemorrage	*Chamaedorea angustisecta*	toasted in infusin	flower	Bolivia	Tacana	UMSA et al. 1999, Bourdy et al. 2000, Rengifo 2007
GENITO.	haemorrhage	*Cocos nucifera*	extract	flower	Bolivia	Tacana	UMSA et al. 1999
GENITO.	irregular menses	*Cocos nucifera*	extract	flower	Bolivia	Tacana	UMSA et al. 1999
GENITO.	kidney ailments	*Euterpe precatoria*	decoction, internally	root, fruit, mesocarp, oil	Bolivia, Peru	Tacana	DeWalt et al. 1999, Deharo et al. 2004, AECID 2007, Rengifo 2007
GENITO.	penis enlarger	*Socratea exorrhiza*	mashed	root	Bolivia, Peru	Tacana	DeWalt et al. 1999, UMSA et al. 1999, Bourdy et al. 2000, Rengifo 2007
GENITO.	kidney ailments	*Phytelephas macrocarpa*		leaf, young	Brazil		Campos and Ehringhaus 2003

GENITO.	diuretic	*Syagrus comosa*		fruit, mesocarp	Brazil		[Pio Correa 1926 nv], Plotkin and Balick 1984
GENITO.	diuretic	*Copernica prunifera*	dried	root	Brazil, Ceará		[Braga 1960 nv, LeCointe 1934 nv], Plotkin and Balick 1984, Balick 1989
GENITO.	diuretic	*Syagrus picrophylla*	fermented beverage	fruit, mesocarp	Brazil, Ceará		[Braga 1960 nv], Plotkin and Balick 1984
GENITO.	renal afflications	*Cocos nucifera*	herbal mixture with *Bambusa vulgaris*, decoction, internally	root	Cuba		Cano and Volpato 2004
GENITO.	vaginal flows	*Cocos nucifera*	decoction herbal mixtures, orally	root	Cuba		Cano and Volpato 2004
GENITO.	prostate hyperplasia	*Roystonea regia*	lipid extract	fruit	Cuba		Arruzazabala et al. 2004, 2005, Gamez et al. 2005, Menedez et al. 2007
GENITO.	impotence	*Roystonea regia*	decoction in herbal mixtures	root	Cuba		Cano and Volpato 2004
GENITO.	renal afflications	*Roystonea regia*	decoction in herbal mixtures, internally	root	Cuba		Cano and Volpato 2004
GENITO.	vaginal flows	*Roystonea regia*	decoction herbal mixtures, orally	root	Cuba		Cano and Volpato 2004
GENITO.	diuretic	*Cocos nucifera*	infusion	fruit, mesocarp	Ecuador	Afroecuatorian	Torre et al. 2007
GENITO.	kidney ailments	*Cocos nucifera*		fruit	Ecuador	Kichwa	Torre et al. 2007
GENITO.	haemorrhage	*Euterpe precatoria*	cooked	root	Ecuador	Kichwa	Lescure et al. 1987, Torre et al. 2007
GENITO.	diuretic	*Phytelephas aequatorialis*	cooked	root	Ecuador	Afroecuatorian	Lescure et al. 1987, Barfod 1991, Torre et al. 2007
GENITO.	haemorrhage	*Cocos nucifera*	boiled with palm heart of *Euterpe oleracea*	root, eastward growing	French Guyana	Arawak	Andel 2000a,b
GENITO.	haemorrhage	*Euterpe oleracea*	boiled with root of *Cocos nucifera*	palm heart, sap	French Guyana	Arawak	Andel 2000a,b

GENITO.	female ailments	*Sabal mauritiiformis*			Panama	Cuna	Johnson, 1999
GENITO.	kidney ailiments	*Astrocaryum chambira*			Peru, Amazon		Rengifo 2007
GENITO.	diuretic	*Lepidocaryum tenue*			Peru, Amazon		Rengifo 2007
GENITO.	diuretic	*Phytelephas macrocarpa*		root	Peru, Ecuador		Duke and Vasquez 1994, Rengifo 2007, Torre et al. 2007
GENITO.	haemorrhage	*Euterpe oleracea*			Peru, Lower Amazon		Rengifo 2007
GENITO.	kidney ailments	*Euterpe oleracea*			Peru, Lower Amazon		Rengifo 2007
GENITO.	dysmenorrhoea	*Euterpe precatoria*			Peru, Lower Amazon		Rengifo 2007
GENITO.	diuretic response in cases of nephritisis and in atrophic cirrosis with ascites	*Cocos nucifera*					Grimwood 1975
GENITO.	diuretic	*Phoenix dactylifera*					Johnson, 1999
GENITO.	kidney ailments	*Sabal sp.*					Johnson, 1999
GENITO.	urinary symptoms	*Serenoa repens*	extract	fruit			Comhaire and Mahmoud, 2004, Gong and Gerber, 2004, Hostanska et al. 2007, Lopatkin et al. 2007
GENITO.	prostate hyperplasia	*Serenoa repens*		fruit			Habib and Wyllie, 2004, Belostotskaia et al. 2006, Beckert et al. 2007, Hizli and Uygur 2007
ILLDEF.	prone to illness, fortificant	*Euterpe precatoria*	general tonic or "milk"	root, fruit mesocarp	Bolivia	Tacana	Bourdy et al. 2000, Deharo et al. 2004

ILLDEF.	debility	*Oenocarpus bataua*			Bolivia		UMSA et al. 1999
ILLDEF.	tonic	*Mauritia flexuosa*	extracted juice	stem, young	Brazil		[Braga 1969 nv], Plotkin and Balick 1984
ILLDEF.	tonic, invigorating	*Mauritiella aculeata*	soaked in water	fruit	Brazil		[Pio Correa 1926 nv], Plotkin and Balick 1984
ILLDEF.	paralysis	*Raphia vinifera*	oil massage	fruit, mesocarp	Brazil		[LeCointe 1934 nv], Plotkin and Balick 1984
ILLDEF.	tonic	*Syagrus oleracea*	eaten fresh	palm heart	Brazil		[Pio Correa 1926 nv.], Plotkin and Balick 1984
ILLDEF.	medicinal	*Attalea butyracea*	fermented sweet sap	stem	Colombia		[Perez Arbelaez 1956 nv], Plotkin and Balick 1984
ILLDEF.	medicinal	*Syagrus cocoines*	fermented into a wine	stem, sap	Colombia		[Perez Arbelaez 1956], Plotkin and Balick 1984
ILLDEF.	Ill defined symptoms	*Bactris corossilla*			Ecuador	Shuar	Torre et al. 2007
ILLDEF.	Ill defined symptoms	*Bactris gasipaes*			Ecuador	Shuar	Torre et al. 2007
ILLDEF.	Ill defined symptoms	*Bactris setulosa*			Ecuador		Torre et al. 2007
ILLDEF.	Ill defined symptoms	*Bactris simplicifrons*			Ecuador		Torre et al. 2007
ILLDEF.	Ill defined symptoms	*Euterpe precatoria*		root	Ecuador	Secoya	Torre et al. 2007
ILLDEF.	Ill defined symptoms	*Geonoma cuneata*		leaf	Ecuador	Awa	Torre et al. 2007
ILLDEF.	Ill defined symptoms	*Geonoma cuneata*		stem	Ecuador	Awa	Torre et al. 2007

ILLDEF.	debility	*Geonoma stricta*		leaf	Ecuador	Huaorani	Torre et al. 2007
ILLDEF.	Ill defined symptoms	*Hyospathe elegans*			Ecuador	Kichwa	Torre et al. 2007
ILLDEF.	Ill defined symptoms	*Iriartea deltoidea*		palm heart	Ecuador	Shuar	Torre et al. 2007
ILLDEF.	Ill defined symptoms	*Mauritia flexuosa*			Ecuador		Torre et al. 2007
ILLDEF.	Ill defined symptoms	*Oenocarpus bataua*			Ecuador	Kichwa	Torre et al. 2007
ILLDEF.	Ill defined symptoms	*Phytelephas tenuicaulis*		root	Ecuador	Secoya	Torre et al. 2007
ILLDEF.	Ill defined symptoms	*Prestoea schultzeana*			Ecuador	Secoya	Torre et al. 2007
ILLDEF.	youthfull	*Coccothrinax argentea*			Haiti		Johnson, 1999
ILLDEF.	debility	*Pritchardia hillebrandii*			Hawaii		Johnson, 1999
ILLDEF.	debility	*Elaeis guineensis*			Peru, Amazon		Rengifo 2007
ILLDEF.	debility	*Cocos nucifera*	jelly from	root	West Indies		Ayensu, 1981
ILLDEF.	analeptic	*Phoenix dactylifera*					Johnson, 1999
ILLDEF.	shaking	*Aiphanes ulei*	decoction, internally	adventitious root	Ecuador	Huaorani	Macia 2004
INFLAM	inflammations of the uterus and ovaries	*Attalea speciosa*		fruit, mesocarp	Brazil		Pereira da Silva and Paz Parente, 2001
INFLAM.	bronchitis	*Attalea phalerata*	internally	fruit, oil	Bolivia	Tacana	DeWalt et al. 1999
INFLAM.	bronchitis	*Attalea speciosa*	internally and friction	fruit, seed, oil	Bolivia	Tacana	UMSA et al. 1999

INFLAM.	antioxidant	*Euterpe precatoria*	aqueous and methanolic extract ot the dried	root	Bolivia		[Desmarchelier et al.. 1997] Deharo et al.. 2004
INFLAM.	bronchitis	*Oenocarpus bataua*	internally	fruit, mesocarp, seed oil	Bolivia, Colombia	Tacana, Llanos	Balick 1988, García Barriga 1974, DeWalt et al. 1999, Bourdy et al. 2000, Bennett et al. 2002
INFLAM.	catarrh	*Acrocomia aculeata*		fruit, endosperm	Brazil		[Pittier 1926 nv], Plotkin and Balick 1984
INFLAM.	inflamations	*Attalea speciosa*		fruit, mesocarp	Brazil		Nascimento et al. 2005
INFLAM.	colitis	*Attalea speciosa*		fruit, mesocarp	Brazil		Pereira da Silva and Paz Parente 2001
INFLAM.	anti-inflammatory	*Attalea speciosa*		fruit, mesocarp	Brazil		Pereira da Silva and Paz Parente, 2001
INFLAM.	allergy	*Phytelephas macrocarpa*		leaf, young	Brazil	Yawanawa	Campos and Ehringhaus 2003
INFLAM.	gout	*Raphia vinifera*	massage	fruit, mesocarp,oil	Brazil		[LeCointe 1934 nv], Plotkin and Balick 1984
INFLAM.	acne of skin	*Euterpe oleracea*		fruit	Brazil NE		Matheus et al. 2006
INFLAM.	stomach inflammation	*Elaeis guineensis*		fruit, oil	Colombia		[Duke 1968 nv], Plotkin and Balick 1984
INFLAM.	pneumonia	*Cocos nucifera*	decoction in herbal mixtures	root	Cuba		Cano and Volpato, 2004
INFLAM.	pneumonia	*Roystonea regia*	decoction in herbal mixtures	root	Cuba		Cano and Volpato 2004
INFLAM.	bronchitis	*Phoenix dactylifera*			Dominican Republic and Haiti		Johnson, 1999
INFLAM.	inflamations	*Bactris gasipaes*		stem, sap of young palm	Ecuador	Shuar	Torre et al. 2007
INFLAM.	bronchitis	*Oenocarpus bataua*		fruit, oil	Ecuador	Kichwa	Torre et al. 2007
INFLAM.	inflamations of	*Phytelephas aequatorialis*		fruit	Ecuador	Tsa'chi	Torre et al. 2007

	ovaries						
INFLAM.	bronchitis	*Bactris oligoclada*	decoction	palm heart	Guyana	Kidalebanaro	[Fanshawe 1950 nv], Plotkin and Balick 1984
INFLAM.	bronchitis	*Euterpe precatoria*	decoction mixed with roots of *Manicaria saccifera* and bamboo leaves	root	Guyana	Winamoru	[Fanshawe 1950 nv], Plotkin and Balick 1984
INFLAM.	bronchitis	*Manicaria saccifera*	decoction	root	Guyana	Winamoru	[Fanshawe 1950 nv], Plotkin and Balick 1984
INFLAM.	bronchitis	*Thrinax radiata*			Mexico		Johnson, 1999
INFLAM.	bronchitis	*Oenocarpus bataua*	mixed with sweet almond oil taken orally or injected	fruit, mesocarp oil	Peru		[Garcia Barriga 1974], Plotkin and Balick 1984
INFLAM.	antioxidant	*Aiphanes horrida*			Peru, Amazon		Rengifo 2007
INFLAM.	surgical wounds	*Attalea speciosa*		fruit, mesocarp			Amorim et al. 2006, Baldez et al. 2006, Martins et al. 2006, Batista et al. 2006
INFLAM.	anti-inflammatory	*Euterpe oleracea*					Matheus et al. 2003, 2006
INFLAM.	anti-inflammatory	*Serenoa repens*	dried	fruit			Balick 1989
INJUR.	to remove foreign body	*Astrocaryum murumuru*	to remove spine or firewood	spine (big one)	Bolivia	Tacana	UMSA et al. 1999
INJUR.	cicatrizant	*Attalea phalerata*	externally, poultice	fruit, seed oil	Bolivia	Tacana	DeWalt et al. 1999, UMSA et al. 1999, Bourdy et al. 2000
INJUR.	snakebite	*Chamaedorea angustisecta*	mashed external	leaf bud	Bolivia	Tacana	UMSA et al. 1999, Bourdy et al. 2000
INJUR.	sting ray	*Chamaedorea angustisecta*	mashed	leaf bud	Bolivia	Tacana	UMSA et al.. 1999, Bourdy et al. 2000
INJUR.	cicatrizant	*Oenocarpus bataua*	externally, poultice	fruit, seed oil	Bolivia	Tacana	UMSA et al. 1999, DeWalt et al.

							1999
INJUR.	snakebite	*Socratea exorrhiza*	mashed	root	Bolivia	Tacana	Bourdy et al. 2000
INJUR.	wounds	*Socratea exorrhiza*	compress	root	Bolivia	Tacana	UMSA et al. 1999, Bourdy et al. 2000
INJUR.	snakebite	*Chamaedorea angustisecta*		flower	Bolivia, Madidi	Tacana	Paniagua-Zambrana 2005
INJUR.	unguents and plasters	*Copernicia prunifera*		leaf, wax	Brazil		[Pio Correa 1926 nv], Plotkin and Balick 1984
INJUR.	boils, bleeding	*Desmoncus mitis*		root	Brazil	Kaxinawa	Campos and Ehringhaus 2003
INJUR.	hemostatic	*Elaeis guineensis*		leaf, axilar hairs	Brazil		[Pio Correa 1926 nv], Plotkin and Balick 1984
INJUR.	hemostatic	*Euterpe edulis*		stem, sap from young	Brazil		[Pio Correa 1926 nv], Plotkin and Balick 1984
INJUR.	hunting remedy	*Geonoma sp.*		fruit	Brazil	Kaxinawa	Campos and Ehringhaus 2003
INJUR.	sting ray	*Syagrus coronata*	extracted, eaten	fruit, seed oil	Brazil		[Pio Correa 1926 nv], Plotkin and Balick 1984
INJUR.	wounds	*Elaeis guineensis*		fruit, oil	Brazil NE		Campos and Ehringhaus 2003
INJUR.	cicatrizant	*Cocos nucifera*		fruit, endosperm	Brazil, Ecuador	Afroecuatorian	Campos and Ehringhaus 2003, Torre et al. 2007
INJUR.	snakebite	*Astrocaryum ciliatum*		stem, sap	Colombia	Miraña	Kahn and Millán, 1992
INJUR.	snakebite	*Astrocaryum sciophilum*	internally	stem, sap	Colombia	Miraña	Galeano 1992, La Rotta et al.. 1989
INJUR.	snakebite	*Bactris guineensis*	decoction	fruit	Colombia		[Garcia Barriga 1974 nv], Plotkin and Balick 1984, Balick 1989, García Barriga 1992
INJUR.	snakebite	*Chamaedorea sp.*			Colombia		Perez Arbelaez 1978, Plotkin and

							Balick 1984
INJUR.	ant bite	*Socratea exorrhiza*	rubbed on skin to calm down burning sensation	root	Colombia	Miraña	Galeano 1992
INJUR.	snakebite	*Bactris guinensis*		fruit	Ecuador		Torre et al. 2007
INJUR.	snakebite	*Socratea exorrhiza*	mashed, infusion and externally	palm heart	Ecuador	Shuar	Byg 2002
INJUR.	scorpion sting	*Euterpe oleracea*			French Guyana	Arawak, Warao	Andel 2000a,b
INJUR.	scorpion stings, to soothe	*Euterpe edulis*	roasted and applied externally and taken internally	palm heart	Guyana		[Fanshawe 1950 nv], Plotkin and Balick 1984
INJUR.	heat	*Cocos nucifera*		root	Jamaica, Exuma and Long Islands and Bahamas		Ayensu, 1981
INJUR.	sting ray	*Iriartea deltoidea*	juce taken internally	root	Peru		AECID 2007
INJUR.	wounds	*Iriartea deltoidea*	externally scrape from	stem	Peru		AECID 2007
INJUR.	snakebite antidote	*Oenocarpus bataua*	infusion	seedlings	Peru	Bora	Plotkin and Balick 1984, Balick 1988
INJUR.	scorpion sting	*Phytelephas macrocarpa*	smoke from burned	leaf	Peru		AECID 2007
INJUR.	sting ray	*Socratea exorrhiza*		root, juice	Peru		AECID 2007
INJUR.	sprain and fracture	*Astrocaryum murumuru*	paste	fruit	Suriname		[May 1965 nv], Plotkin and Balick 1984
INJUR.	hemostatic	*Euterpe oleracea*	liquid dripped into cuts	stem, sap	Suriname, Guyana	Matowai, Arawak, Warao	Plotkin and Balick 1984, Andel 2000a,b
INJUR.	snakebite	*Acrocomia aculeata*		root	Trinidad and Tobago		Lans et al. 2001

INJUR.	antiseptic	*Attalea speciosa*	liquid expressed from rachis	leaf			Balick 1986
MENTAL	anti-hysteric	*Syagrus oleracea*	eaten fresh	palm heart	Brazil		[Pio Correa 1926 nv], Plotkin and Balick 1984
MENTAL	tranquilizer	*Bactris gasipaes*		palm heart of young palm	Ecuador	Shuar	Torre et al. 2007
MENTAL	soporific	*Bactris sp. (Balick #1050)*	ripe fruits consumed in large quantities	fruit, endosperm	Peru	Bora	Plotkin and Balick 1984
MENTAL	prevent sleep	*Euterpe oleracea*					Balick 1986
MUSCUL.	chest complaints	*Attalea phalerata*	raw or fried, rubbed on the affected part of the body	stems, beetle larvae living in decaying trunks	Bolivia	Tacana	DeWalt et al. 1999
MUSCUL.	rheumatism	*Attalea speciosa*	liniment	fruit, endosperm	Brazil		[Pio Correa 1926 nv], Plotkin and Balick 1984, Silva and Parente, 2001, Nascimento et al. 2005
MUSCUL.	arthritis	*Attalea speciosa*		fruit, mesocarp	Brazil		Pereira da Silva and Paz Parente, 2001
MUSCUL.	rheumatism	*Attalea speciosa*		fruit, mesocarp	Brazil		Silva and Parente, 2001, Nascimento et al. 2005
MUSCUL.	rheumatism	*Bactris gasipaes*		fruit, seed oil	Brazil		[Pio Correa 1926 nv], Plotkin and Balick 1984, Balick 1989
MUSCUL.	arthrits	*Cocos nucifera*	decoction	fruit, husk fiber	Brazil		Esquenazi et al. 2002
MUSCUL.	rheumatism	*Raphia vinifera*	oil massage	fruit, mesocarp	Brazil		[LeCointe 1934 nv], Plotkin and Balick 1984
MUSCUL.	arthritis	*Copernicia prunifera*		root	Brazil, Ceará		[Braga 1960 nv], Plotkin and Balick 1984
MUSCUL.	rheumatism	*Copernicia prunifera*		root	Brazil, Ceará		[Braga 1960 nv], Plotkin and Balick 1984

MUSCUL.	arthritis	*Leopoldinia pulchra*	soaked in alcohol	leaf	Colombia	Tukano	Schultes and Raffauf 1990
MUSCUL.	chest complaints	*Astrocaryum chambira*	raw or fried, rubbed on the affected part of the body	stems, beetle larvae living in decaying trunks	Ecuador	Huaorani	Ceron and Ayala 1998
MUSCUL.	artrithis	*Oenocarpus bataua*		fruit, oil	Ecuador	Kichwa	Torre et al. 2007
MUSCUL.	rheumatism	*Oenocarpus bataua*		fruit, oil	Ecuador	Kichwa	Torre et al. 2007
MUSCUL.	chest complaints	*Wettinia maynensis*	raw or fried, rubbed on the affected part of the body	stems, beetle larvae living in decaying trunks	Ecuador	Huaorani	Ceron and Ayala 1998
MUSCUL.	arthritic inflammataions	*Oenocarpus bataua*	mixed with liniment and rubbed	fruit, oil	Peru		[Balick 1980 nv], Plotkin and Balick 1984, Balick 1989
MUSCUL.	rheumatism	*Astrocaryum chambira*			Peru, Amazon		Rengifo 2007
MUSCUL.	chest complaints	*Oenocarpus bataua*	raw or fried rubbed on the affected part of the body	beetle larvae living in decaying stem	Peru, Ecuador, Bolivia	Bora, Cayaps, Coaiqueres, Huaorani, Tacana	[Balick 1980] Plotkin and Balick 1984, Barford and Balslev 1988, Ceron and Ayala 1998, DeWalt et al. 1999, UMSA et al. 1999, Macia 2004
MUSCUL.	rheumatism	*Elaeis oleifera*			Peru, Lower Amazon		Rengifo 2007
MUSCUL.	sprains and fractures	*Astrocaryum murumuru*	paste	fruit	Suriname		Plotkin and Balick 1984
MUSCUL.	sprains and fractures	*Astrocaryum sciophilum*	paste	fruit	Suriname		Plotkin and Balick 1984
NEOPL.	leukemia, tumor, ulcer	*Attalea speciosa*		fruit, mesocarp	Brazil		Silva and Parente, 2001, Nascimento et al. 2005
NEOPL.	leukemia	*Cocos nucifera*		fruit, husk fiber	Brazil		Koshek 2007

NEOPL.	cancer	*Phoenix dactylifera*			Dominican Republic and Haiti		Johnson, 1999
NEOPL.	tumour	*Desmoncus giganteus*			Ecuador	Kichwa	Torre et al. 2007
NEOPL.	cancer	*Sabal mexicana*			Mexico		Johnson, 1999
NEOPL.	cancer	*Aiphanes horrida*		fruit, seed	Peru		Lee et al. 2001, Rengifo 2007
NEOPL.	cancer	*Cocos nucifera*	extract				Esquenazi et al. 2002
NEOPL.	prostate cancer, prevention	*Serenoa repens*					Comhaire and Mahmoud 2004, Habib et al. 2005, Wadsworth et al. 2007
NEUROL.	epilepsy	*Phytelephas macrocarpa*		leaf, young	Brazil	Yawanawa	Campos and Ehringhaus 2003
NUTRIT.	obesity	*Attalea speciosa*		fruit, mesocarp	Brazil		Silva and Parente, 2001, Nascimento et al. 2005
NUTRIT.	nutritive "strengthening those who are weak due to old age"	*Mauritia flexuosa*	fermented beverage called "Chicha de canangua"	fruit	Colombia		[Schultes 1951 nv], Plotkin and Balick 1984, Schultes and Raffauf 1990
NUTRIT.	scorbut	*Roystonea regia*			Haiti		Johnson, 1999
NUTRIT.	scorbut	*Cocos nucifera*			West Indies		Ayensu, 1981
NUTRIT.	malnutrition	*Cocos nucifera*		fruit, endosperm liquid			Grimwood, 1975
ODONT.	dental care	*Hyospathe elegans*	chewing	palm heart	Colombia	Witoto	Galeano 1992
ODONT.	caries, prevention	*Geonoma stricta*	chew	palm heart	Ecuador	Siona	Torre et al. 2007
ODONT.	dental care	*Hyospathe elegans*	fresh	palm heart	Ecuador	Siona	Lescure et al. 1987
ODONT.	dental care	*Hyospathe elegans*	ash	leaf	Ecuador	Siona-Secoya	Schultes and Raffauf 1990
ODONT.	dental care	*Chamaedorea pinnatifrons*	chewed to blacken teeth	leaf, petiole	Peru	Mayna Jivaro	Schultes and Raffauf 1990

ODONT.	caries, prevention	*Bactris gasipaes*			Peru, Amazon		Rengifo 2007
PAIN	headache	*Attalea phalerata*	externally	fruit, oil	Bolivia	Tacana	DeWalt et al. 1999, Bourdy et al. 2000
PAIN	kidney pain	*Attalea phalerata*	internally and extrernally	fruit, seed oil	Bolivia	Tacana	UMSA et al. 1999, Bourdy et al. 2000
PAIN	headache	*Attalea speciosa*	massage	fruit, seed, oil	Bolivia	Tacana	UMSA et al. 1999
PAIN	abdominal pain	*Chamaedorea angustisecta*	infusion, internally	flower	Bolivia	Tacana	Bourdy et al. 2000
PAIN	liver pain	*Euterpe precatoria*	decoction, internally	root	Bolivia	Tacana	UMSA et al. 1999, Bourdy et al. 2000
PAIN	stomach ache	*Geonoma pohliana*	infusion	flower	Bolivia	Tacana	UMSA et al. 1999, Bourdy et al. 2000
PAIN	liver pain	*Oenocarpus bataua*	internally	fruit, oil	Bolivia	Tacana	UMSA et al. 1999, Bourdy et a. 2000
PAIN	body pain	*Oenocarpus bataua*	internally and massage	fruit, oil	Bolivia	Tacana	UMSA et al. 1999, Bourdy et al. 2000
PAIN	stomach ache	*Bactris acanthocarpa*	boiled in water, decoction cooled is drunk	fruit	Bolivia, Beni	Chacobo	Boom 1987
PAIN	chest pain	*Euterpe precatoria*	shreded and boiled pinnae, cooled decoction is drunk	leaf	Bolivia, Beni	Chacobo	Boom 1987
PAIN	stomach ache	*Chamadorea angustisecta*	infusion, internally	flower	Bolivia, Peru	Tacana	UMSA et al. 1999, Rengifo 2007
PAIN	muscular, back or sciatic pain	*Euterpe precatoria*	decoction or syrup	root	Bolivia, Peru	Tacana	Deharo et al. 2004, Rengifo 2007
PAIN	kidney pain	*Euterpe precatoria*	decocotion, internally	root	Bolivia, Peru	Tacana	UMSA et al. 1999, Bourdy et al. 2000, Diaz 2003
PAIN	menstrual pain	*Attalea phalerata*		fruit, mesocarp	Brazil		Silva and Parente, 2001, Nascimento et al. 2005

PAIN	pain	*Attalea speciosa*		fruit, mesocarp	Brazil		Nascimento et al. 2005
PAIN	headache	*Phytelephas macrocarpa*		leaf, young	Brazil	Yawanawa, Kaxinawa	Campos and Ehringhaus 2003
PAIN	pain	*Euterpe oleracea*			Brazil NE		Matheus et al. 2006
PAIN	sprain pain	*Mauritiella aculeata*	boiled from the ripe fruits rubbed warm on the affected muscle	fruit	Colombia	Puinave	Schultes and Raffauf 1990
PAIN	stomach ache	*Phoenix dactylifera*			Dominican Republic and Haiti		Johnson, 1999
PAIN	body pain	*Aiphanes ulei*	decoction, internally	root (adventitious)	Ecuador	Huaorani	Macia 2004
PAIN	headache	*Aiphanes ulei*	decoction, internally	root (adventitious)	Ecuador	Siona, Huaorani	Macia 2004, Torre et al. 2007
PAIN	body pain	*Aiphanes ulei*		palm heart	Ecuador	Siona	Torre et al. 2007
PAIN	stomach ache	*Ammandra decasperma*	internally	fruit, endosperm	Ecuador	Huaorani	Torre et al. 2007
PAIN	stomach ache	*Bactris corossilla*	mixed with chili peppers	palm heart	Ecuador	Huaorani	Ceron and Ayala 1998
PAIN	earache	*Bactris gasipaes*	warm extract added to ear	palm heart	Ecuador	Shuar	Broseghini and Frucci, 1986, Byg 2002, Bennett et al. 2002
PAIN	throatache	*Bactris maraja*	decoction	root (adventitious)	Ecuador	Huaorani	Macia 2004, Torre et al. 2007
PAIN	body pain	*Desmoncus mitis*	decoction, internally	stem, crushed pinnae	Ecuador	Huaorani	Macia 2004
PAIN	stomach ache	*Desmoncus orthacanthos*		root	Ecuador	Siona	Torre et al. 2007
PAIN	muscular pain	*Euterpe precatoria*	cooked	root	Ecuador	Kichwa	Lescure et al. 1987, Torre et al. 2007
PAIN	throatache	*Euterpe precatoria*	decoction mixed with mother's milk	root (adventitious)	Ecuador	Huaorani	Macia 2004, Torre et al. 2007
PAIN	bump pain	*Geonoma cuneata*	macerated and heated	stem	Ecuador	Cachi	Torre et al. 2007

PAIN	sprain pain	*Geonoma macrostachys*		leaf	Ecuador	Kichwa	Torre et al. 2007
PAIN	headache	*Oenocarpus bataua*	internally juce mixed with yuca	root adventitious	Ecuador	Huaorani	Plotkin and Balick 1984, Lescure et al. 1987, Schultes and Raffauf 1990, Kahn and Granville 1992, Russo 1992, Macia 2004, Torre 2007
PAIN	body pain	*Oenocarpus bataua*		fruit, oil	Ecuador	Kichwa	Torre et al. 2007
PAIN	stomach ache	*Oenocarpus bataua*	liquid mixed with yuca	fruit, adventitious root	Ecuador	Huaorani	Torre et al. 2007
PAIN	throatache	*Prestoea schultzeana*	decoction	root (adventitious)	Ecuador	Huaorani	Macia 2004, Torre et al. 2007
PAIN	knee ache	*Socratea exorrhiza*	boiled and applied as a poultice	root (growing stilt)	Ecuador	Huaorani	Macia 2004, Torre et al. 2007
PAIN	toothache	*Cocos nucifera*		root	Jamaica, Exuma and Long Islands and Bahamas		Ayensu, 1981
PAIN	anodyne	*Oenocarpus bataua*		fruit, mesocarp oil	Panama	Darien	[Duke 1968 nv], Plotkin and Balick 1984
PAIN	eye ache	*Phytelephas macrocarpa*	aplied to the eye	fruit, liqiud	Peru		AECID 2007
PAIN	headache	*Elaeis guineensis*			Peru, Amazon		Rengifo 2007
PAIN	back pain	*Astrocaryum murumuru*	decoction, internally	root	Peru, Bolivia	Tacana	UMSA et al. 1999, Bourdy et al. 2000, Rengifo 2007
PAIN	headache	*Oenocarpus bataua*	externally	fruit, oil	Peru, Bolivia	Tacana	Rengifo 2007, DeWalt et al. 1999
PAIN	menstrual pain	*Calamus sp.*	tea	leaf, flower	West Indies		Ayensu, 1981
PAIN	toothache	*Astrocaryum vulgare*		fruit, seed oil		Palikur	Kahn and Millán, 1992
PAIN	antinociceptive	*Cocos nucifera*	aqueous extract	fruit, husk fiber			Alviano et al. 2004

PAIN	stomach ache	*Desmoncus polyacanthos*		fruit		Apinayé	Balick 1986
PAIN	antinociceptive	*Euterpe oleracea*					Marinho et al., 2003
PAIN	stomach ache	*Geonoma pohliana*	tea	leaf, young		Apinayé	Balick 1986
PAIN	menstrual pain	*Attalea speciosa*		fruit, mesocarp	Brazil		Pereira da Silva and Paz Parente 2001
PARASI.	intestinal infection	*Attalea phalerata*	decoction, internally	root, fruit, oil	Bolivia	Tacana	DeWalt et al. 1999, UMSA et al. 1999, Bourdy et al. 2000
PARASI.	leishmaniasis	*Socratea exorrhiza*	mashed used externally	root	Bolivia, Peru	Tacana	DeWalt et al. 1999, Bourdy et al. 2000, Rengifo 2007
PARASI.	scabies	*Socratea exorrhiza*	compress	root	Bolivia, Peru	Tacana	UMSA et al. 1999, Bourdy et al. 2000, Rengifo 2007
PARASI.	jigger, skin ectoparasite	*Attalea oleifera*		fruit, seed oil	Brazil		[Pereira 1929 nv], Plotkin and Balick 1984
PARASI.	helminthiasis	*Butia yatay*		fruit, endosperm	Brazil		[Pio Correa 1926 nv], Plotkin and Balick 1984,
PARASI.	taenifuge	*Astrocaryum aculeatissimum*		fruit, dried endosperm oil	Brazil, Ecuador	Huaorani	[Pio Correa 1926, nv], Plotkin and Balick 1984, Torre et al. 2007
PARASI.	helminthiasis	*Astrocaryum aculeatissimum*		fruit, mesocarp oil	Brazil, Sao Paulo		[Pereira 1929 nv], Plotkin and Balick 1984
PARASI.	helminthiasis	*Bactris guineensis*	decoction	fruit	Colombia		Garcia Barriga 1974, Plotkin and Balick 1984
PARASI.	helminthiasis	*Cocos nucifera*	raw or decoction	fruit, endosperm, mesocarp	Colombia		Garcia Barriga 1974
PARASI.	helminthiasis	*Elaeis guineensis*		fruit	Colombia		Garcia Barriga 1974
PARASI.	malaria	*Euterpe sp.*	decoction	root	Colombia	Tikuna	Schultes and Raffauf 1990
PARASI.	helminthiasis	*Cocos nucifera*		fruit, oil	Cuba		Ayensu 1981, Cano and Volpato

							2004
PARASI.	helminthiasis	*Cocos nucifera*	decoction, juice extraction, internally	fruit	Cuba		Cano and Volpato 2004
PARASI.	helminthiasis	*Bactris corossilla*	internally	palm heart	Ecuador	Huaorani	Ceron and Ayala 1998
PARASI.	helminthiasis	*Cocos nucifera*		fruit, seed	Ecuador		Acosta Solis, 1992
PARASI.	helminthiasis	*Cocos nucifera*		fruit, endosperm	Ecuador	Afroecuatorian	Torre et al. 2007
PARASI.	parasitic infections	*Wettinia maynensis*		palm heart	Ecuador	Kichwa	Torre et al. 2007
PARASI.	helminthiasis	*Oenocarpus bataua*		root adventitious	Ecuador, Peru	Huaorani	Plotkin and Balick 1984, Schultes and Raffauf 1990, Kahn and Granville 1992, Rengifo 2007, Torre et al. 2007
PARASI.	helminthiasis	*Acrocomia aculeata*			Mexico	Mixe	Leonti et al. 2003
PARASI.	helminthiasis	*Cocos nucifera*		fruit, exocarp	Mexico		Alanís et al 2005
PARASI.	trichomoniasis	*Cocos nucifera*	infusion, internally	fruit, husk fiber	Mexico		Calzada et al. 2007
PARASI.	malaria	*Euterpe edulis*	decoction, drunk	root	Peru	Shipibo-Conibo	Milliken 1997
PARASI.	malaria	*Euterpe precatoria*	infusion, internally	root	Peru		AECID 2007, Rengifo 2007
PARASI.	malaria	*Oenocarpus mapora*	mashed taken internally	fruit green	Peru		[Duke, Vasquez 1994] Milliken, Rengifo 2007
PARASI.	malaria	*Oenocarpus minor*	mashed taken internally	fruit green	Peru		Duke, Vasquez 1994
PARASI.	helminthiasis	*Astrocaryum chambira*			Peru, Amazon		Rengifo 2007
PARASI.	malaria	*Oenocarpus bataua*			Peru, Amazon		Rengifo 2007

PARASI.	intestinal infection	*Phytelephas macrocarpa*			Peru, Amazon		Rengifo 2007
PARASI.	malaria	*Phytelephas macrocarpa*			Peru, Amazon		Rengifo 2007
PARASI.	malaria	*Euterpe oleracea*			Peru, Lower Amazon		Rengifo 2007
PARASI.	worms from dogs	*Dypsis lutescens*			Trinidad and Tobago		Ayensu, 1981
PARASI.	helminthiasis	*Astrocaryum aculeatissimum*		fruit, endosperm			Balick 1989
PARASI.	helminthiasis	*Bactris gasipaes*		root			Mora-Uprí et al. 1997
PARASI.	helminthiasis	*Cocos nucifera*	itself or mixed with coconut milk	fruit, endosperm, liquid			Grimwood 1975
PARASI.	schistosomiasis	*Cocos nucifera*		fruit, coconut water			Hirose 2005
PARASI.	leishmaniasis, topical application in wound healing	*Cocos nucifera*	polyphenolic-rich extract				Mendonca-Filho et al. 2004
PARASI.	skin parasites	*Astrocaryum aculeatum*	spines used to remove	spine	Venezuela	Yanomami	Gertsch et al., 2002
POISON.	antbite	*Iriartea deltoidea*	squized sprouts are put pnto the spot that hurts	leaf	Venezuela	Yanomami	Gertsch et al., 2002
POISON.	antbite	*Socratea exorrhiza*	squized sprouts are put pnto the spot that hurts	leaf	Venezuela	Yanomami	Gertsch et al., 2002
PARASI.	antiplasmodial	*Euterpe precatoria*		root			Jensen et al. 2002
POISON.	insect repellent	*Attalea princeps*	burned on charcoal	fruit, husk fiber	Bolivia		Moor 2007
POISON.	ichthyotoxin	*Socratea exorrhiza*	mashed and thrown in water	root	Bolivia	Tacana	DeWalt et al. 1999
POISON.	insect repellent	*Elaeis guineensis*	anointment	fruit, oil	Brazil		[Pio Correa 1926 nv], Plotkin and Balick 1984

POISON.	snakebite	*Euterpe precatoria*		leaf (young)	Brazil	Yawanawa, Kaxinawa	Campos and Ehringhaus 2003
POISON.	curare antidote	*Leopoldinia major*	ash applied directly to the wound	fruit	Brazil		[LeCointe 1934 nv], Plotkin and Balick 1984, Lorenzi H. 1996
POISON.	snakebite	*Phytelephas macrocarpa*		leaf, young	Brazil	Yawanawa, Kaxinawa	Campos and Ehringhaus 2003
POISON.	sting ray	*Phytelephas macrocarpa*		leaf, young	Brazil	Kaxinawa	Campos and Ehringhaus 2003
POISON.	curare admixture	*Socratea exorrhiza*	exudate from	root	Brazil	Mayongong	Plotkin and Balick 1984
POISON.	piscicide	*Syagrus cocoides*		flower	Brazil, Maranhao		[Hoehne 1939 nv], Plotkin and Balick 1983
POISON.	poisonous to people	*Syagrus cocoides*		fruit	Brazil, Maranhao		[Hoehne 1939 nv], Plotkin and Balick 1984
POISON.	ichthyotoxin	*Bactris sp*	ash aded to Caryocar fish poison	leaf	Colombia	Kubeo	Schultes and Raffauf 1990
POISON.	poison to kill people	*Geonoma macrostachys*	decoction with other plants	palm heart	Ecuador	Kichwa	Balslev et al. 1997
POISON.	curare admixture	*Geonoma stricta*	ashes	leaf	Ecuador	Kofan	Schultes and Raffauf 1990
POISON.	ichthyotoxin	*Sabal palmetto*	eaten or steep in gin for a drink	palm heart	Exuma and Long Islands and Bahamas		Ayensu, 1981
POISON.	poisonous to birds	*Euterpe sp.*		fruit	Venezuela	Warao	[Wilbert 1975 nv], Plotkin and Balick 1984
PREGN.	anticoncepcional and abortive	*Syagrus romanzoffiana*			Argentina		[Toursarkissian 1980], Colares et al. 1997
PREGN.	increase lactation	*Astrocaryum murumuru*		leaf young	Boliva	Tacana	DeWalt et al. 1999
PREGN.	childbirth	*Socratea exorrhiza*	to poultice on umbical cords of newborn babies	grated stipes	Brazil	Wayapi	Duke and Vasquez 1994

PREGN.	pregnancy vomits	*Cocos nucifera*	raw	fruit, endosperm	Colombia		Garcia Barriga 1974
PREGN.	miscarriage, prevention	*Cocos nucifera*	scorched over fire, boiled in water, then drunk	leaf, bases from trunk	Colombia	Tikuna	Schultes and Raffauf 1990
PREGN.	abortive	*Cocos nucifera*	decoction, herbal mixtures	fruit, unripe	Cuba		Cano and Volpato 2004
PREGN.	anticonceptional	*Cocos nucifera*	decoction	fruit, unripe	Cuba		Cano and Volpato 2004
PREGN.	abortive	*Roystonea regia*	decoction, internally	stem, bark	Cuba		Cano and Volpato 2004
PREGN.	increase lactation	*Bactris gasipaes*	cooked and rubbed on the chest	root	Ecuador	Kichwa	Torre et al. 2007
PREGN.	chilldbirth	*Geonoma macrostachys*		fruit	Ecuador	Huaorani	Torre et al. 2007
PREGN.	parturifacient	*Iriartea deltoidea*	inner leaf sheath layer used with *Iris sp.*	leaf, sheath	Ecuador		Plotkin and Balick 1984, Schultes and Raffauf 1990, Bennett et al. 2002
PREGN.	increase lactation	*Mauritia flexuosa*		palm heart	Ecuador		Torre et al. 2007
PREGN.	childbirth	*Astrocaryum gynacanthum*	to dry a newborn baby's navel string ash from	leaf	Guyana	Arawak, Carib	Andel 2000a,b
PREGN.	miscarriage, prevention	*Cocos nucifera*	decoction drunk with a massage	root, fruit	Honduras		Ticktin and Dalle 2005
PREGN.	increase conception	*Acrocomia aculeata*	fermented into a wine	sap	Venezuela		[Pittier 1926, nv] Plotkin and Balick 1984, Balick 1989
PREGN.	infant feeding	*Cocos nucifera*		fruit, endosperm, liquid			Grimwood 1975
PREGN.	childbirth	*Oenocarpus bataua*	infant's mouth cleaned by oil-soaked cloth	fruit, mesocarp, oil			[Balick 1979 nv], Plotkin and Balick 1984, Balick 1986
PREGN.	anticoncepcional and abortive	*Phoenix dactylifera*					Johnson, 1999
RESPIR.	cough	*Attalea phalerata*	decoction, internally	fruit, oil	Bolivia	Tacana	DeWalt et al. 1999, UMSA et al. 1999

RESPIR.	cough	*Attalea speciosa*	internally and friction	fruit, seed, oil	Bolivia	Tacana	UMSA et al. 1999
RESPIR.	pulmonary ailments	*Attalea phalerata*	internally and extrernally	fruit, seed oil	Bolivia, Madidi	Tacana	Bourdy et al. 2000, Paniagua-Zambrana 2005
RESPIR.	pulmonary ailments	*Attalea speciosa*		fruit, seed, oil	Bolivia, Madidi		Paniagua-Zambrana 2005
RESPIR.	cough	*Euterpe precatoria*		leaf (young)	Brazil	Yawanawa	Campos and Ehringhaus 2003
RESPIR.	asthma	*Oenocarpus mapora*		fruit, oil	Brazil		Campos and Ehringhaus 2003
RESPIR.	pectoral	*Syagrus romanzoffiana*	macerated in wine or liquid made into syrup	fruit	Brazil		[Pio Correa 1926 nv], Plotkin and Balick 1984
RESPIR.	asthma	*Oenocarpus bataua*		fruit, oil	Brazil, Ecuador	Kichwa	Campos and Ehringhaus 2003, Torre et al. 2007
RESPIR.	pulmonary ailments	*Astrocaryum aculeatum*	rubbed warm on the chest	fruit, seed oil	Colombia	Puinaves	Schultes and Raffauf 1990
RESPIR.	congestion	*Astrocaryum gynacanthum*	inhale smoke from burned	leaf	Colombia	Witoto	Schultes and Raffauf 1990
RESPIR.	asthma	*Manicaria saccifera*	milk extract	fruit	Colombia		Perez-Arbelaez 1978
RESPIR.	catarrh	*Oenocarpus bataua*		fruit, mesocarp oil	Colombia	Llanos	Garcia Barriga 1974
RESPIR.	breath purifier	*Phoenix dactylifera*	dried or dissolved	fruit	Colombia		García Barriga 1974
RESPIR.	pectoral	*Acrocomia aculeata*		flower, after dropping	Colombia, Antioquia		Perez-Arbelaez 1978, Plotkin and Balick 1984
RESPIR.	pulmonary ailments	*Oenocarpus bataua*	internally	fruit, mesocarp, oil	Colombia, Bolivia, Peru	Llanos, Tacana	Plotkin and Balick 1984, UMSA et al. 1999, Bourdy et al. 2000, Paniagua-Zambrana 2005, Rengifo 2007
RESPIR.	asthma	*Cocos nucifera*	juice extraction and maceration in herbal mixtures	fruit	Cuba		Cano and Volpato 2004
RESPIR.	phlegm	*Cocos nucifera*	extract, internally	fruit, juice	Cuba		Cano and Volpato 2004

RESPIR.	asthma	*Roystonea regia*	frying, trituration in herbal mixtures	fruit	Cuba		Cano and Volpato 2004
RESPIR.	cough	*Aiphanes ulei*	internally decoction of crushed	palm heart	Ecuador	Huaorani	Macia 2004, Torre et al. 2007
RESPIR.	pulmonary ailments	*Attalea phalerata*	raw or fried, rubbed on the chest	stems, beetle larvae living in decaying trunks	Ecuador	Cayapas, Coaiqueres	Barford and Balslev 1988
RESPIR.	cough	*Bactris concinna*	decoction, internally	root (adventitious)	Ecuador	Huaorani	Macia 2003, Torre et al. 2007
RESPIR.	cough	*Bactris maraja*	decoction, internally	root	Ecuador	Huaorani	Macia 2003, Torre et al. 2007
RESPIR.	cough	*Geonoma deversa*	chewed	fruit	Ecuador	Huaorani	Macia 2004
RESPIR.	cough	*Oenocarpus bataua*	liquid mixed with yuca	root (adventitious)	Ecuador	Huaorani	Macia 2003, Torre et al. 2007
RESPIR.	cough	*Prestoea schultzeana*	decoction, internally	root (adventitious)	Ecuador	Huaorani	Macia 2004, Torre et al. 2007
RESPIR.	cough	*Euterpe precatoria*	decoction mixed with mother's milk	root (adventitious)	Ecuador, Peru	Huaorani	Macia 2003, Rengifo 2007, Torre et al. 2007
RESPIR.	asthma	*Oenocarpus bataua*		palm heart	Ecuador, Peru		Bennett et al. 2002, Rengifo 2007
RESPIR.	cough	*Manicaria saccifera*	"milk" from young	fruit, root	Guyana	Warao	[Pittier 1926 nv], Plotkin and Balick 1984
RESPIR.	cough	*Oenocarpus mapora*	infusion, internally	fruit	Peru		AECID 2007
RESPIR.	cough	*Oenocarpus bataua*	mixed with sweet almond oil	fruit, oil	Peru, Colombia		[Garcia Barriga 1974 nv], Plotkin and Balick 1984
RESPIR.	breath purifier	*Astrocaryum vulgare*		fruit, mesocarp	Suriname		[May 1965 nv], Plotkin and Balick 1984
RESPIR.	cough	*Astrocaryum vulgare*		fruit, mesocarp	Suriname		[May 1965 nv], Plotkin and Balick 1984
RESPIR.	asthma	*Manicaria saccifera*	milk extract	fruit, root	Venezuela, Guyana	Warao	[Pittier 1926 nv], Plotkin and Balick 1984

RESPIR.	congestion	*Manicaria saccifera*	water of	fruit		Warao	Plotkin and Balick 1984
RESPIR.	asthma	*Phoenix dactylifera*					Johnson, 1999
SENSOR	ophthalmia	*Attalea princeps*	extract from ripening	fruit, endosperm, liquid	Brazil		[Pio Correa 1926 nv], Plotkin and Balick 1984
SENSOR	eye irritation	*Syagrus microphylla*	liquid	fruit, endosperm	Brazil		Henderson et al. 1995
SENSOR	ophthalmia	*Syagrus schizophylla*	extract from ripening	fruit, liqiud	Brazil		[Pio Correa 1926 nv], Plotkin and Balick 1984
SENSOR	conjunctivitis	*Phytelephas macrocarpa*	aplied to the eye	fruit, liqiud	Brazil, Peru		AECID 2007
SENSOR	eye infection	*Allagoptera campestris*	juice	fruit, still green	Brazil, Sao Paulo		[Pereira 1929 nv], Plotkin and Balick 1984
SENSOR	eye infection	*Lepidocaryum tenue*	roasted and squeezed	stem	Peru	Bora	Balick 1989, Rengifo 2007
SENSOR	eye ailments	*Sabal sp.*				Houma	Johnson, 1999
SKIN	hair care	*Attalea princeps*		fruit, mesocarp oil	Bolivia		[Cardenas 1969 nv], Plotkin and Balick 1984
SKIN	dandruff	*Attalea speciosa*	applied 1-2 daily	fruit, oil	Bolivia		Balick 1989
SKIN	pimple, skin ichting	*Socratea exorrhiza*	mashed used externally	root	Bolivia	Tacana	Bourdy et al. 2000
SKIN	skin ulcerations	*Socratea exorrhiza*	compress	root	Bolivia	Tacana	UMSA et al. 1999
SKIN	hair care	*Attalea phalerata*		fruit, endosperm, oil	Bolivia, Brazil	Tacana, Yawanawa, Kaxinawa	[Cardenes 1969] Plotkin and Balick 1984, DeWalt et al. 1999, Campos and Ehringhaus 2003
SKIN	hair care	*Astrocaryum murumuru*	extract	fruit, oil	Brazil	Yawanawa, Kaxinawa	Campos and Ehringhaus 2003
SKIN	baldness	*Attalea princeps*		fruit, endosperm oil	Brazil		[Pio Correa 1926 nv], Plotkin and Balick 1984
SKIN	hair care	*Attalea tessmannii*		fruit, oil	Brazil	Yawanawa	Campos and Ehringhaus 2003

SKIN	rashes	*Attalea tessmannii*		fruit, oil	Brazil	Yawanawa	Campos and Ehringhaus 2003
SKIN	skin ailments e.g. eczema	*Desmoncus orthacanthos*		root	Brazil		[Pio Correa 1926 nv], Plotkin and Balick 1984, Balick 1989
SKIN	hair care	*Euterpe precatoria*	extract	fruit, oil	Brazil	Yawanawa, Kaxinawa	Campos and Ehringhaus 2003
SKIN	emollient	*Mauritia flexuosa*	added to baths	leaf	Brazil		[Braga 1960 nv], Plotkin and Balick 1984
SKIN	hair care	*Oenocarpus bataua*		fruit, oil	Brazil	Kaxinawa, Yawanawa	Campos and Ehringhaus 2003
SKIN	rashes	*Oenocarpus bataua*		fruit, oil	Brazil	Yawanawa	Campos and Ehringhaus 2003
SKIN	hair care	*Oenocarpus mapora*		fruit, oil	Brazil	Yawanawa, Kaxinawa	Campos and Ehringhaus 2003
SKIN	rashes	*Oenocarpus mapora*		fruit, oil	Brazil	Yawanawa, Kaxinawa	Campos and Ehringhaus 2003
SKIN	emollient	*Oenocarpus minor*	extract green oil	fruit, mesocarp	Brazil		[Pereira 1929 nv], Plotkin and Balick 1984
SKIN	causes severe skin ichting	*Socratea exorrhiza*		root (adventitious)	Brazil	Mayongong	Plotkin and Balick 1984
SKIN	emollient	*Oenocarpus distichus*	extract green oil	fruit, mesocarp	Brazil Sao Paulo		[Pereira 1929 nv], Plotkin and Balick 1984
SKIN	skin ailments	*Copernicia prunifera*		root	Brazil, Ceará		[Braga 1960 nv], Plotkin and Balick 1984, Balick 1989
SKIN	dandruff	*Elaeis guineensis*	hair tonic	fruit, oil	Brazil, Colombia		[Pio Correa 1926 nv, Usher 1974 nv], Plotkin and Balick 1984
SKIN	dandruff	*Elaeis oleifera*	hair tonic	fruit, oil	Brazil, Colombia		Plotkin and Balick 1984
SKIN	relapse of	*Elaeis guineensis*	extract	fruit, oil	Colombia		Garcia Barriga 1974

	dermatosis						
SKIN	delousing	*Iriartella setigera*	externally, macerated	leaf	Colombia		Galeano 1992
SKIN	delousing	*Wettinia augusta*	externally	leaf	Colombia	one Muinane speaking informant	Galeano 1992
SKIN	wart	*Aiphanes ulei*	externaly	fruit	Ecuador	Kichwa	Torre et al. 2007
SKIN	delousing	*Chamadorea pauciflora*	tied to the forehead	inflorescence	Ecuador	Huaorani	Macia 2004
SKIN	astringent	*Cocos nucifera*		flower, root	Ecuador	Afroecuatorian	Torre et al. 2007
SKIN	spots	*Geonoma stricta*		fruit	Ecuador	Huaorani	Torre et al. 2007
SKIN	skin infections	*Hyospathe elegans*			Ecuador	Kichwa	Lescure et al. 1987
SKIN	spots	*Hyospathe elegans*			Ecuador	Kichwa	Torre et al. 2007
SKIN	beneficial to the skin	*Cocos nucifera*		fruit, seed oil	Guyana	Arawak, Carib, Warao	Andel 2000a
SKIN	baldness	*Bactris gasipaes*			Peru, Amazon		Rengifo 2007
SKIN	hair, skin tonic	*Bactris gasipaes*			Peru, Amazon		Rengifo 2007
SKIN	edema	*Elaeis guineensis*			Peru, Amazon		Rengifo 2007
SKIN	spots	*Socratea exorrhiza*			Peru, Amazon		Rengifo 2007
SKIN	baldness	*Euterpe oleracea*			Peru, Lower Amazon		Rengifo 2007
SKIN	nettle rash	*Chamaedorea fragrans*			Peru, Upper Amazon		Rengifo 2007

SKIN	boils	*Astrocaryum vulgare*		fruit, seed oil		Palikur	Kahn and Millán, 1992
SKIN	furunculosis	*Astrocaryum vulgare*		root		Palikur	Kahn and Millán, 1992
SKIN	baldness	*Cocos nucifera*		fruit, endosperm oil			Acosta Solis, 1992
SKIN	xerosis	*Cocos nucifera*	moistuizer	fruit, coconut oil, coconut water			Agero and Verallo-Rowell, 2004, Grimwood 1975
SKIN	ulcers, sores	*Cocos nucifera*	outside shining part of	fruit			Ayensu, 1981
SKIN	edema	*Euterpe precatoria*					Deharo et al. 2004
SKIN	skin ulcerations	*Euterpe precatoria*					Deharo et al. 2004
SKIN	baldness	*Oenocarpus bataua*		fruit, oil		Quichua	Gallegos 1988, Balick 1988, DeWalt et al. 1999, Bennett et al. 2002
SKIN	sun protection	*Oenocarpus bataua*		fruit, oil		Boras, Witotos, Andokes, Ocaina, Yagua, Tikunas	Balick 1986
SKIN	emollient	*Phoenix dactylifera*					Johnson, 1999
SOCIAL	ambil-syrup for coca chewing	*Attalea insignis*	ash	root	Colombia	Andokes	Schultes and Raffauf 1990
SOCIAL	bewitched	*Attalea luetzelburgii*	"little men" live in plant	inflorescence, spathe	Colombia	Kubeo	Schultes and Raffauf 1990
SOCIAL	tobacco syrup admixture	*Geonoma deversa*	ash	leaf	Colombia	Witotos	Schultes and Raffauf 1990
SOCIAL	ambil-syrup admixture	*Hyospathe elegans*	ashes evaporated in water	leaf	Colombia	Witoto	Schultes and Raffauf 1990, Bennett et al. 2002
SOCIAL	healing rituals	*Oenocarpus mapora*		leaf	Colombia	Embera	Henderson et al. 1995
SOCIAL	intoxicant	*Pseudophoenix lediniana*			Dominican Republic		Johnson, 1999

SOCIAL	intoxicant	*Phoenix dactylifera*			Dominican Republic, Haiti		Johnson, 1999
SOCIAL	emetic to puryfy the body before taking ayahuasca	*Chamaedorea pauciflora*	infusion		Ecuador	Siona-Secoya	Schultes and Raffauf 1990
SOCIAL	coca admixture, alkaline	*Attalea princeps*	ash	inflorescence, spadix	Peru		Schultes and Raffauf 1990
SOCIAL	coca admixture	*Attalea tessmannii*	ash	stem, bark	Peru	Boras	Schultes and Raffauf 1990
SOCIAL	susto	*Socratea exorrhiza*	walk among supporting roots and eat after bruise banana to exert vomiting	root	Peru	Ashaninka	own obs. Sosnowska 2008
SOCIAL	intoxicant	*Attalea speciosa*			Venezuela		Johnson, 1999
SOCIAL	wrapping sacred tobacco cigars	*Euterpe sp*		leaf, stipule	Venezuela	Warao	[Wilbert 1975 nv], Plotkin and Balick 1984
SOCIAL	intoxicant	*Syagrus amara*			West Indies		Johnson, 1999
VIRAL	jaundice	*Cocos nucifera*		fruit, endosperm, liquid	Bahamas, Jamaica, Brazil		Ayensu 1981, Campos and Ehringhaus 2003
VIRAL	measles	*Oenocarpus bataua*	internally, externally	fruit, oil	Bolivia	Tacana	Bourdy et al. 2000
VIRAL	smallpox	*Oenocarpus bataua*	internally, externally	fruit, oil	Bolivia	Tacana	UMSA et al. 1999
VIRAL	cold	*Euterpe oleracea*		fruit, juice	Brazil NE		Matheus et al. 2006
VIRAL	jaundice	*Astrocaryum aculeatissimum*	liquid from unripe	fruit	Brazil, Ecuador	Huaorani	[Pio Correa 1926, nv], Plotkin and Balick 1984, Torre et al. 2007
VIRAL	jaundice	*Euterpe precatoria*	infusion, internally	root	Brazil, Peru	Kaxinawa	Campos and Ehringhaus 2003, AECID 2007, Rengifo 2007
VIRAL	influenza	*Euterpe precatoria*	mixed with *Hyospathe elegans*	root	Colombia	Miraña	Galeano 1992

			palm heart				
VIRAL	influenza	*Hyospathe elegans*	mixed with *Euterpe precatoria* root	palm heart	Colombia	Miraña	Galeano 1992
VIRAL	cold	*Mauritia flexuosa*	fermented beverage	fruit	Colombia		Schultes and Raffauf 1990
VIRAL	influenza	*Mauritia flexuosa*	fermented beverage	fruit	Colombia		Schultes and Raffauf 1990
VIRAL	cold	*Oenocarpus bataua*	internally or injected	fruit, mesocarp oil	Colombia	Llanos	Garcia Barriga 1974
VIRAL	influenza	*Oenocarpus bataua*	internally or injected		Colombia	Llanos	Plotkin and Balick 1984, García Barriga 1992, Bennett et al. 2002
VIRAL	cold	*Aiphanes ulei*	internally decoction of crushed	palm heart	Ecuador	Huaorani	Macia 2004
VIRAL	cold	*Aiphanes ulei*	decoction, internally	root (adventitious)	Ecuador	Huaorani, Siona	Macia 2004, Torre et al. 2007
VIRAL	influenza	*Aiphanes ulei*	cooked	palm heart	Ecuador	Huaorani	Torre et al. 2007
VIRAL	measles	*Astrocaryum chambira*		fruit	Ecuador	Cofan	Cerón Martinez 1995, Torre et al. 2007
VIRAL	cold	*Astrocaryum urostachys*	internally	fruit, endosperm	Ecuador	Huaorani	Macia 2004, Torre et al. 2007
VIRAL	cold	*Bactris concinna*	decoction, internally	root (adventitious)	Ecuador	Huaorani	Macia 2004, Torre et al. 2007
VIRAL	cold	*Bactris maraja*	cooked	root	Ecuador	Huaorani	Torre et al. 2007
VIRAL	cold	*Desmoncus mitis*	internally decoction of crushed	stem, pinnae	Ecuador	Huaorani	Macia 2004
VIRAL	influenza	*Desmoncus mitis*	decoction	stem, pinnas	Ecuador	Huaorani	Torre et al. 2007
VIRAL	influenza	*Euterpe precatoria*	internally decoction mixed with mother's milk	leaf, root	Ecuador	Huaorani	Ceron and Ayala 1998, Torre et al. 2007
VIRAL	cold	*Euterpe precatoria*	internally juice from chewed or crushed	root (adventitious)	Ecuador	Huaorani	Macia 2004

VIRAL	influenza	*Geonoma deversa*		fruit	Ecuador	Huaorani	Torre et al. 2007
VIRAL	holanda	*Geonoma polyandra*	ash	leaf	Ecuador	Siona	Torre et al. 2007
VIRAL	influenza	*Oenocarpus bataua*	liquid mixed with yucca	fruit, oil, root (adventitous)	Ecuador	Kichwa, Huaorani	Ceron and Ayala 1998, Torre et al. 2007
VIRAL	cold	*Oenocarpus bataua*	internally juice mixed with yucca	root (adventitious)	Ecuador	Huaorani	Macia 2004
VIRAL	cold	*Oenocarpus bataua*	internally after boiling	fruit, mesocarp	Ecuador	Huaorani	Macia 2004
VIRAL	cold	*Prestoea schultzeana*	decoction, internally	root (adventitious)	Ecuador	Huaorani	Macia 2004
VIRAL	influenza	*Prestoea schultzeana*	decoction	root	Ecuador	Huaorani	Torre et al. 2007
VIRAL	cold	*Attalea maripa*	infusion	fruit	Ecuador, Peru	Huaorani	Plotkin and Balick 1984, Lescure et al. 1987, Balick 1989, Schultes and Raffauf 1990, Bennett et al. 2002, Torre et al. 2007, Rengifo 2007
VIRAL	jaundice	*Astrocaryum chambira*			Peru, Amazon		Rengifo 2007
VIRAL	jaundice	*Iriartella setigera*			Peru, Amazon		Rengifo 2007
VIRAL	jaundice	*Phytelephas macrocarpa*			Peru, Amazon		Rengifo 2007
VIRAL	jaundice	*Socratea exorrhiza*	decoction	leaf	Peru, Amazon	Achual	Duke and Vasquez 1994, Rengifo 2007
VIRAL	jaundice	*Euterpe oleracea*			Peru, Lower Amazon		Rengifo 2007
VIRAL	cold in chest	*Cocos nucifera*		fruit, oil			Ayensu 1981
VIRAL	antiviral activity	*Cocos nucifera*	crude extract	fruit, husk fiber			Esquenazi et al. 2002
VIRAL	rashes from small-pox, chicken-pox,	*Cocos nucifera*					Grimwood 1975

	measles						
VIRAL	jaundice	*Oenocarpus distichus*	boiled in water, cooled, taken once a day for three days	fruit, mesocarp		Apinayé	Balick 1986
	intravenous infusions	*Cocos nucifera*	withdrawn with the aid of a tube inserted throug an eye. The fluid is then filtred to drain off any particles of	coconut water			[Science News Letter 1954], Grimwood 1975

List of abbreviations used in Table 1
own obs. = own observation
BACTER. - bacterial infections, BLOOD - blood system disorders, CIRCUL. -circulatory system disorders, DIGEST. - digestive system disorders, ENDOCR. - endocrine system disorders, FUNGAL - fungal infections, GENITO. – genitourinary system disorders, ILLDEF. - ill-defined symptoms, INFLAM. - inflammations, INJUR. - injuries, MENTAL - mental disorders, MUSCUL. - muscular-skeletal system disorders, NEUROL. - neurological system disorders, NEOPL. - neoplasms, NUTRIT. - nutritional disorders, ODONT. - odontological disorders, PARASI. - parasitic infections, POISON. - poisonings, PREGN. - pregnancy/birth/puerperium disorders, RESPIR. - respiratory system disorders, SENSOR. - sensory system
